# Cue the volatility spillover in the cryptocurrency markets during the COVID-19 pandemic: evidence from DCC-GARCH and wavelet analysis

**DOI:** 10.1186/s40854-021-00319-0

**Published:** 2022-02-03

**Authors:** Onur Özdemir

**Affiliations:** Department of International Trade and Finance (English), Istanbul Gelisim University, Istanbul, Turkey

**Keywords:** Volatility spillover, EGARCH, DCC-GARCH, Wavelets, COVID-19, C50, C58, E44

## Abstract

This study investigates the dynamic mechanism of financial markets on volatility spillovers across eight major cryptocurrency returns, namely Bitcoin, Ethereum, Stellar, Ripple, Tether, Cardano, Litecoin, and Eos from November 17, 2019, to January 25, 2021. The study captures the financial behavior of investors during the COVID-19 pandemic as a result of national lockdowns and slowdown of production. Three different methods, namely, EGARCH, DCC-GARCH, and wavelet, are used to understand whether cryptocurrency markets have been exposed to extreme volatility. While GARCH family models provide information about asset returns at given time scales, wavelets capture that information across different frequencies without losing inputs from the time horizon. The overall results show that three cryptocurrency markets (i.e., Bitcoin, Ethereum, and Litecoin) are highly volatile and mutually dependent over the sample period. This result means that any kind of shock in one market leads investors to act in the same direction in the other market and thus indirectly causes volatility spillovers in those markets. The results also imply that the volatility spillover across cryptocurrency markets was more influential in the second lockdown that started at the beginning of November 2020. Finally, to calculate the financial risk, two methods—namely, value-at-risk (VaR) and conditional value-at-risk (CVaR)—are used, along with two additional stock indices (the Shanghai Composite Index and S&P 500). Regardless of the confidence level investigated, the selected crypto assets, with the exception of the USDT were found to have substantially greater downside risk than SSE and S&P 500.

## Introduction

Triggered by the recent rapid rise in the price of cryptocurrencies such as Bitcoin, Ethereum, and Litecoin, a lively interest in research on these explosive bubbles has emerged as to whether the COVID-19 pandemic stimulated risky behaviors among financial investors. The major concern of this debate is that the digital asset markets have neither intrinsic value nor offer dividends. In the last couple of years, the cryptocurrency markets have launched an exponential increase in the demand for those assets, and thereby have witnessed a huge growth in the degree of market capitalization (Kumar and Ajaz 2019). According to White (2015), the total number of cryptocurrencies tripled from 2014 to 2018. However, a very recent investigation from one of the most cited financial platforms, CoinMarketCap, shows that the total number of cryptocurrencies is 6955 as of September 9, 2020, which covers a market capitalization of approximately $325 billion. In this vein, the huge growth in market capitalization and the expanding number of digital assets lead us to predict potential problems that may occur along with an increase in market liquidity. More importantly, the greater expected returns on asset gains may reinforce some legal impediments to curtail financial inclusion that induce financial fragility and promote financial instability. Considering this possible outcome and gaining momentum in these markets, some crucial points and important aspects of cryptocurrency markets should be listed in proper sequence, as they may result in a growing scale of financial disturbance across the globe.

Starting from the COVID-19 outbreak, the cryptocurrency market has seen investors earn abnormal returns in cryptocurrencies, implying a growing inefficiency. Compared to traditional financial assets, cryptocurrencies are exposed to extreme volatility. Depending on the relative volatility in the cryptocurrency market, Böhme et al. (2015) refer to this kind of market structure being shallow since, in such a market, any downturn may suddenly trigger a market crash. Factors such as the lack of regularity or tax policy framework in cryptocurrency markets also tend to persistence in high volatility, thus raise the emergence of financial fraud. According to Vasek and Moore (2015), the estimated loss from various scams and fraudulent activities in cryptocurrency markets is approximately $11 million. This is due to the possible unequal distribution of assets in cryptocurrency markets, where first-generation miners and investors have more financial power to buy related assets than the other generations, exacerbated by volatility spillovers (Smith and Kumar 2018). In this regard, the size and structure of cryptocurrency markets have been affected by price movements (Gandal et al. 2018).

As the historical events of the COVID-19 pandemic reveal, many investors lost a large amount of their income due to factors such as the slowdown of production, net erosion in the value of financial assets and real assets, competitive depreciation, the breakdown of supply chains, ongoing problems in the payment of past debts, and the reduction of consumption levels. While these factors can be extended to micro- and macro-based contexts, the overall outlook on the economic functions of the COVID-19 pandemic shows that the problems do not only emerge from the real sector but also from the financial sector. In consideration of these mixed and complicated issues, many investors lacked the money to maintain their purchasing of stocks, but most importantly, they also avoided investing in stocks and bonds due to uncertainty in the future. Therefore, they tended to transfer their financial resources to cryptocurrency markets where there was a chance to obtain the potential of outsized returns to compensate for their current losses. In addition, the unique freedom of that market made investors want to invest in such cryptocurrencies to hedge their losses during the COVID-19 pandemic. This financial transfer into the cryptocurrency markets can also be understood as a pathbreaking shift in the financial era since none of the pre-COVID-19 periods exhibited such behavior in which the financial investors substantially moved toward those market caps. Hence, the hedging features of cryptocurrencies might also stimulate the emergence of crisis-led dynamics for further periods due to a surge in volatile behaviors.

This paper focuses on this issue by appplying different complimentary methods to grasp cryptocurrency market dynamics during the COVID-19 outbreak. The use of these different models is based on theoretical considerations for detecting the different time scales of volatility transmission across eight digital assets. First, the presence of volatility spillovers in cryptocurrency markets was analyzed using the exponential GARCH (EGARCH) model. Second, the DDC model was applied, which allows conditional correlations to vary over time (Engle 2002). This model follows the flexibility of the univariate GARCH models. However, it simplifies parameterization and estimation (Sabkha and de Peretti 2018). Finally, wavelet methods were used to analyze spillover effects through various time scales. One of the main reasons for the application of wavelet-based procedures was the fractal market hypothesis (Peters 1994), where financial markets follow a cyclical and iterative pattern, implying that investors act independently over time. In addition, the GARCH framework does not fully grasp the information of all time scales, even though it controls the conditional correlation and covariances. Following the theoretical background of Kumar and Anandarao (2019), the empirical strategy utilized the DCC-GARCH and wavelet methods to provide estimates of various frequencies without losing information from the selected time horizon. The EGARCH model estimations were produced via EViews 10, and the DCC-GARCH model and wavelet-based method estimations were obtained using Microfit 5 and RStudio software, respectively.

While this paper focuses on three different but integrated methods, there are also many studies that use different techniques to show that the volatility spillovers in the cryptocurrency markets are still prevalent. These methods can be classified as follows: rolling sample analysis (Fasanya et al. 2021), BEKK-MGARCH analysis (Katsiampa et al. 2019b), machine learning techniques (e.g., linear models, random forests, and support vector machines) (Sebastião and Godinho 2021), an integrated cluster detection, optimization, and interpretation approach (Li et al. 2021), Markov regime-switching vector autoregressive with exogenous variables (MS-VARX) model (Shahzad et al. 2021), generalized VAR framework (Melki 2020), bankruptcy prediction model (Kou et al. 2021a, b), a hybrid interval type-2 fuzzy multidimensional decision-making approach (Kou et al. 2021a, b), and multivariate stochastic volatility model (Zhang and He 2021). The common point of using these methods is to capture the inherent secular and cyclical movements in digital asset markets. However, all of these findings emphasize the importance of information loss obtained from the returns of assets. Therefore, this study uses the DCC-GARCH and wavelet methods to provide estimates of various frequencies without losing information from the selected time horizon, which also critically differs from the above methods.

As it is binding for the financial sector, an even higher rate of volatility spillover may also be possible for digital currencies such as cryptocurrencies, which may be much more than the rest of the others. While many assets are controlled by rules and policies, cryptocurrencies are not tied to a bank or government, thereby allowing investors to spend money anonymously. The coins are materialized by investors who mine them by lending electronic power to affirm other investors’ transactions in which the cryptocurrencies are bought in exchange. Therefore, considering the lack of control mechanisms and corporate infrastructure, cryptocurrencies encounter several potential threats when giant investors sell their assets, resulting in a substantial decrease in prices. While this provides a net gain for investors who have a chance to actively control and monitor the market, the rest of the small investors are confronted by a net loss when prices drop below the buying price. This then paves the way for giant investors to invest in those markets to stimulate the demand mechanism along with an increase in the prices of assets, thereby providing speculation on unbounded and unrestrained digital currencies. According to several laureates of the Nobel Memorial Prize in Economic Sciences (e.g., Paul Krugman, Robert J Shiller, Joseph Stiglitz, Richard Thaler, James Heckman, Thomas Sargent, Angus Deaton, and Oliver Hart), central bankers (e.g., Alan Greenspan, Agustin Carstens, Vitor Constâncio, and Nout Wellink), and investors (Warren Buffett, George Soros, Jack Ma, and Jamie Dimon), most of the major cryptocurrencies are speculative bubbles. For instance, Patterson (2018) argues that cryptocurrencies are highly prone to severe bubbles. He examines at real-world data such as the fact that the crypto lost 80 percent of its value, on average, which was greater than the bursting of the dotcom bubble in 2002. Moreover, Huang (2018) and Meyer (2018) note that the total market capitalization for Bitcoin fell below $100 billion for the first time in November 2018. In addition, Wilson (2020) argues that Bitcoin plummets as cryptocurrencies suffer in market turmoil by looking at the data in which the price of Bitcoin fell by 30% from $8901 to $6206 in only four days from March 8 to March 12, 2020. Furthermore, Bitcoin’s price increased over 700% from March 2020 to early 2021 and reached above $61,000 for the first time during the COVID-19 pandemic. The UK Financial Conduct Authority warned investors against lending or investing in crypto assets, an suggested that that they should be prepared “to lose all their money”. The historical dynamics of the boom and crash were also evident in the last surge in prices of Bitcoin and Ethereum, when both of these assets dropped in values by 30% and 40% by mid-May 2021, respectively. Major cryptocurrency exchanges were ruined amid a market-wide price crash as a result of Elon Musk’s announcement and the announcement from the People’s Bank of China. All of these examples show that the inner dynamics of crypto assets with a lack of institutional control mechanisms and a lack of policy formation are prone to these severe threats, known as “speculative bubbles” in recent literature.

One of the core differences between cryptocurrency markets and other financial markets is the limit on the number of transactions. While the supply of traditional currencies is under the control of monetary authorities, the number of digital assets in circulation is processed independently. In other words, this non-authorized peer-to-peer payment network is used in a decentralized manner, and therefore, no single person or group has control of the blockchain system, which is assumed to be the digital ledger of transactions. However, the lack of authority in the supply of digital assets and the increasing rates of their returns have raised questions about the safety and stability of cryptocurrency markets. In the case of its potential to lead to financial instability, one of the most common ways to reduce the fast-growing cryptocurrency market is government-issued notices. The other most influential feature of the legislation is the issue of taxation (Marian 2013; Molloy 2019; Solodan 2019; OECD 2020; Renwick and Gleasure 2020). However, the challenge of the taxation process appears to be how to classify different types of cryptocurrencies along with their trading activities. Many questions arise from the case of cryptocurrency taxation where mining or selling cryptocurrencies is classified under the heading of income or capital gains.

Regarding volatility spillovers in the cryptocurrency market, the empirical results provided by the majority of studies indicate that information inefficiency is of primary importance (Urquhart and Hudson 2013; Ito et al. 2014; Urquhart and McGroarty 2016; Hu et al. 2019; Kyrazis 2019; Le Tran and Leirvik 2020). Related to the evidence of inefficiency, other studies show that cryptocurrencies are mutually linked in their feedback positions reflected by volatility spillover, volatility co-movement, lead/lag effect, calendar effect, systematic risk, and day-of-the-week effect (Aharon and Qadan 2019; Omane-Adjepong and Alagidede 2019; Canh et al. 2019; Katsiampa et al. 2019a; Palamalai and Maity 2019; Sifat et al. 2019; Yuneline 2019; Yousaf and Ali 2020; Corbet et al. 2021; Ghorbel and Jeribi 2021; Kinateder and Papavassiliou 2021). Systematic issues of regulatory oversight, the potential for illicit use, and infrastructural breaches have also been thoroughly investigated by Corbet et al. (2019). According to Le Tran and Leirvik (2020), the cryptocurrency market is subject to a certain level of difficulty to trade in conditions of low liquidity, which leads to an astonishing rise in risk and inefficiency. The ease of trading among different cryptocurrencies is largely exposed to variations in the level of liquidity (Phillip et al. 2019). Hence, it has garnered interest from many researchers because of the close linkage between liquidity and market efficiency (Leirvik et al. 2017; Wei 2018; de la Horra et al. 2019; Noda 2020; Takaishi and Adachi 2020; Zhang et al. 2020). Furthermore, cryptocurrencies show a particular type of behavior, such as long memory and multifractality (Bariviera et al. 2017; Al-Yahyaee et al. 2018; Derbentsev et al. 2019; Mensi et al. 2019; Bariviera 2020).

As cryptocurrencies are highly volatile compared to traditional financial assets, attention should be given to a number of studies on the possibility of the market being more liquid. A glance at the current literature on volatility spillovers shows that two strands of empirical evidence suggest conditional volatility in the presence of a generalized autoregressive conditional heteroskedasticity (GARCH) framework and dynamic conditional correlation (DCC) models (Kristoufek 2015; Katsiampa 2017; Urquhart 2017; Trabelsi 2018; Kumar and Anandarao 2019; Liu and Serletis 2019; Bouri et al. 2021). In such models, the presence of volatility spillovers in cryptocurrency markets is of crucial importance. However, ongoing debate on this phenomenon in the existing literature is limited and rare.

Overall, the primary motivation of this study is to determine whether cryptocurrency markets are exposed to volatilities in terms of highly active digital assets. In this vein, the research question is grounded in the questions of how and which channels of influence affected the behavior of investors in cryptocurrency markets during the slowdown in the real sector at the time of the COVID-19 pandemic. Therefore, the proposed contribution of this paper to the literature is to introduce that financial investors may take part in risky behavior in cryptocurrency markets in order to compensate for their losses in assets as a result of the negative effects of the COVID-19 pandemic. This highlights that the overall change in the behavior of investors leads to an increase in volatility in digital asset markets. The remainder of this paper is organized as follows: “Literature review” section provides a brief review of the existing literature. “Data and methodology” section summarizes the research data, and explains the methodological framework. “Results and discussion” section presents our empirical results. “Concluding remarks” section concludes.

## Literature review

The rise in the value of cryptocurrencies in the digital asset market has promoted a number of studies aimed at investigating the dynamics of that market along with listing the major influence of channels. Research interests in cryptocurrency markets concentrate on the following issues: (1) accounting and value formation (Bouoiyour et al. 2016; Blau 2017; Hayes 2017; Urquhart 2017; Zhu et al. 2017; Sovbetov 2018; Bolt and Van Oordt 2019; Zimmerman 2020), (2) speculative bubbles, herding behavior, and lottery-like demand (Godsiff 2015; Poyser 2018; Grobys and Junttila 2021); and (3) forecasting cryptocurrency returns, volume, and price (Azari 2019; Bohte and Rossini 2019; Derbentsev et al. 2019; Nasir et al. 2019; Cohen 2020; Mudassir et al. 2020). In addition, other studies focus on the volatility issue in the case of the relationship between the cryptocurrency market and gold and energy instruments (Huynh et al. 2020a, b; Huynh et al. 2020a, b; Thampanya et al. 2020). However, another issue of the volatility spillovers in the cryptocurrency markets that needs further investigation is the changing dynamics of asset behaviors at the outset of the COVID-19 pandemic (Huynh 2019; Conlon and McGee 2020; Umar and Gubareva 2020; Baur and Dimpfl 2021; Corbet et al. 2021).

There are numerous studies in the literature that put forward the argument that the demand for digital assets has been growing over time. In particular, the COVID-19 pandemic caused a rapid rise in the prices of several cryptocurrencies. Consequently, a number of studies examined the potential factors behind a surge in prices of those assets and also to any kind of shocks that might occur in the market due to the emergence of potential speculative motives. Although the literature explains many potential reasons for explosive bubbles in the cryptocurrency market, there is still no consensus on which factors most impacted the surge of asset prices. The main argument of this study is based on two channels. First, it is argued that the COVID-19 pandemic has changed the behavior of financial investors who bought more digital assets relative to stocks or bonds. This has also promoted volatilities in digital asset markets, as a growing number of investors moved away from buying less profitable and inefficient assets other than digital assets. Given the slowdown in production and productivity and the recession in several sectors at the outset of the COVID-19 pandemic, these investors balanced their potential losses by buying more risky assets such as digital assets in the cryptocurrency market. Second the other potential channel is herding behavior in such markets. As an increasing number of investors have been drawn to the cryptocurrency market it has led to a significant increase in the prices of those assets, as other financial investors have been drawn to buy those assets. However, the main problem is the emergence of an astronomical increase in prices, together with an increase in bubble-type behavior and volatility spillover in the cryptocurrency market during the COVID-19 pandemic.

In addition, several studies have discussed the volatility spillovers of digital assets, suggesting that they behave differently than traditional assets. Initial findings suggesting high volatility in Bitcoin returns in comparison with major exchange rates are found in Sapuric and Kokkinaki (2014) even though it is ensued to be stabilized albeit the Bitcoin transactions are hold. Conrad et al. (2018) investigated the long- and short-term volatility components of cryptocurrencies using the GARCH-MIDAS model and found that S&P 500 realized volatility is negatively and highly significantly correlated with long-term Bitcoin volatility. Baur and Dimpfl (2018) studied asymmetric volatility effects for the 20 largest cryptocurrencies by assuming that there is an atypical effect for financial assets at which positive shocks to markets increase volatility more than negative shocks do. In a recent study, Akyildirim et al. (2020) provided evidence that the contagion of significant financial market stress affects newly produced financial tools and instruments. In a similar study, Dyhrberg (2016) found similarities between the Bitcoin volatility framework to gold and the dollar. Naimy and Hayek (2018) using GARCH models found that the nature of Bitcoin differs from traditional currencies, implying that the behaviors might change over time. Pichl and Kaizoji (2017) found that BTC prices are more volatile than the USD/Euro and USD/CNY currency pairs by employing the heterogeneous autoregressive (HAR) model and a neural network model, respectively.

The existing literature shows that comprehensive analysis has been done based on the consideration of capturing information across various frequencies and at continuum time–space.^1^ To express it differently, the current literature is sparse in that the volatility dynamics of the cryptocurrency markets might exhibit a significant change at the outset of the COVID-19 pandemic. One of the core reasons behind this change may be the increasing scale of explosive bubbles due to a high relative return of cryptocurrencies compared with those of other financial assets. Furthermore, other factors behind the changing dynamics of volatility in cryptocurrency markets can be listed as follows: (1) a sway in perceived value, (2) uncertainty about the future, (3) lack of legal protection, (4) tax treatment, and (5) investors’ expectations. Regarding these matters, the volatility dimension of cryptocurrencies may have then transferred outstanding issues to other asset markets toward an increasing scale of explosive behaviors during the COVID-19 pandemic and the future if the market risk premium was falsely perceived by the financial actors.

Considering this possibility, we studied the volatility spillover of the top eight cryptocurrencies (e.g., in terms of their market value) from November 17, 2019 (i.e., the first official announcement of the COVID-19 case by South China Morning) to January 25, 2021, during which the prices of top cryptocurrencies started rising to unexpected levels. Therefore, the potential differences in cryptocurrency markets in terms of time-led variations in investors’ behavior caused is to move away from the time series approach adopted by Kumar and Ajaz (2019). The next section identifies the possible persistence of volatility in cryptocurrency returns by employing the EGARCH (1,1,1) and DCC-GRACH models. We then used wavelet analysis.to estimate the low and high frequencies of any kind of shock within and across markets that may occur due to the operations of several investors at different time scales.

## Data and methodology

This study investigates volatility spillovers in the cryptocurrency markets of the top eight digital assets during the COVID-19 outbreak. The dataset comprises daily closing prices of Bitcoin (BTC), Ethereum (ETH), Stellar (XLM), Ripple (XRP), Tether (USDT), Cardano (ADA), Litecoin (LTC), and Eos (EOS), *P*_t_s, in U.S. dollars during the period from November 17, 2019, to January 25, 2021, extracted from CoinDesk.^2^ Because the cryptocurrencies are exchanged through continuum moments, the data are employed for all available days, and thus it corresponds to a total of *T* = 465 days for the selected cryptocurrencies.

As a primary concern of this section, the three different methods, EGARCH, DCC-GARCH, and wavelet-based models will be explained in their theoretical context to understand the degree of volatility persistence, which may not have been directly observable during the COVID-19 pandemic. First, we start with the EGARCH model (Nelson 1991) used to detect the conditional variance of the closing prices of the selected cryptocurrencies. The EGARCH model is specifically used to capture the leverage effects of shocks (e.g., policy changes, inefficient information, economic incidents, and social events) on financial markets. It allows for testing asymmetries. With any kind of negative shock, financial assets tend to enter a state of turbulence, and thus, volatility decreases. To capture the net effects of shocks, a logarithmic scale of variance was used in the analysis. The specification for the conditional variance for EGARCH (*p*, *q, r*) is obtained as

1 $${\text{log}}(\sigma_{t}^{2} ) = \omega + \mathop \sum \limits_{j = 1}^{q} \eta_{j} \log \left( {\sigma_{t - j}^{2} } \right) + \mathop \sum \limits_{i = 1}^{p} \gamma_{i} \left| {\frac{{\varepsilon_{t - i} }}{{\sigma_{t - i} }}} \right| + \mathop \sum \limits_{k = 1}^{r} \lambda_{k} \frac{{\varepsilon_{t - k} }}{{\sigma_{t - k} }}$$

where the left-hand side represents the *log* of the conditional variance. This means that the leverage effect is exponential rather than quadratic. In this vein, the forecasting of conditional variance ensures that the estimates are non-negative. Moreover, ${\gamma }_{i}<0$ implies that the presence of the leverage effect is relevant, but if ${\gamma }_{i}\ne 0$, the impact will be asymmetric. In other words, if ${\gamma }_{1}= {\gamma }_{2}=\dots =0$ the model will be considered symmetric. Thus, ${\gamma }_{i}<0$ indicates the case in which negative shocks lead to volatility compared to positive shocks. In addition, $\omega$ is a constant, $\eta$ is the ARCH effect, $\gamma$ is the asymmetric effect, and $\lambda$ is the GARCH effect.

The DCC-GARCH model (Engle 2002) was used to address the time-varying volatilities and correlations among various digital assets. In particular, the model allows for a Gaussian distribution, although it might lead to inefficient findings for a heavy-tailed distribution. Therefore, Pesaran and Pesaran (2007) suggest a DDC-GARCH model with a multivariate *t*-distribution. The covariance matrix is expressed as follows:

2 $${H}_{t}={D}_{t}{R}_{t}{D}_{t}$$

where ${D}_{t}=diag\{\sqrt{{h}_{it}}\}$ is a $k x k$ diagonal matrix of time-varying standard deviations on its diagonals issued from the estimation of univariate GARCH processes, and ${R}_{t}$ is the time-varying conditional correlation matrix of the standardized disturbances, ${\varepsilon }_{t}$. Based on Eq. (2), the conditional returns are normally distributed with a zero mean in the DCC model. The conditional variance $({h}_{it})$ for digital assets is measured by employing the univariate GARCH (X, Y) model, which is given in Eq. (3):

3 $$h_{it} = \omega_{i} + \mathop \sum \limits_{x = 1}^{{X_{t} }} \alpha_{ix} r_{it - x}^{2} + \mathop \sum \limits_{y = 1}^{{Y_{t} }} \beta_{iy} h_{it - y} ,\quad for\, i = 1,2, \ldots ,k$$

where ${\alpha }_{ix}$ denotes the short-run persistence of the shocks to asset returns to long-run persistence (i.e., the GARCH effects, λ) with non-negative values, ${\omega }_{i}$ and ${\beta }_{iy}$ are also non-negative, and the number of digital assets is given by $k$. The conditional standard deviations $(\sqrt{{h}_{it}})$ play a crucial role in the case of a diagonal matrix ${D}_{t}$, which includes $(\sqrt{{h}_{it}})$ the elements on its diagonals, as represented in Eq. (4):

4 $$D_{t} = \left[ {\begin{array}{*{20}c} {\sqrt {h_{11,t} } } & 0 & \ldots & 0 \\ 0 & {\sqrt {h_{22,t} } } & \ldots & 0 \\ \vdots & \vdots & \vdots & 0 \\ 0 & 0 & \ldots & {\sqrt {h_{kk,t} } } \\ \end{array} } \right]$$

The standardized residuals, which is ${\sigma }_{it}={\varepsilon }_{it}/\sqrt{{h}_{it}}$, are expressed for measuring the dynamic correlation matrix, ${R}_{t}$:

5 $${R}_{t}={Q}_{t}^{*-1}{Q}_{t}{Q}_{t}^{*-1}$$

where ${Q}_{t}=({q}_{ij,t})$ is a positive definite matrix with the conditional variances-covariances of ${\varepsilon }_{it}$ and ${Q}_{t}^{*-1}$ is the inverted diagonal matrix. ${Q}_{t}^{*}$ represents the diagonal matrix of its diagonal elements.

6 $${Q}_{t}^{*}=\left[\begin{array}{cccc}\sqrt{{q}_{11}}& 0& \dots & 0\\ 0& \sqrt{{q}_{22}}& \dots & 0\\ \vdots & \vdots & \vdots & 0\\ 0& 0& \dots & \sqrt{{q}_{kk}}\end{array}\right]$$

In this regard, the DCC model can be estimated by the unconditional covariance of the standardized distribution $\overline{Q }$, of the univariate GARCH model:

7 $${Q}_{t}=\left(1-a-b\right)\overline{Q }+\alpha {\varepsilon }_{t-1}-1{\varepsilon }_{t-1}^{^{\prime}}{+bQ}_{t-1}$$

The dynamic conditional correlations are then given by:

8 $${p}_{ij,t}=\frac{{q}_{ij,t}}{\sqrt{{q}_{ii,t}{q}_{jj,t}}}$$

According to Engle (2002), the estimations obtained from the DCC model use a two-step maximum likelihood estimation method. Thus, the likelihood function is expressed as

9 $$\ln \left( {L\left( \theta \right)} \right) = - \frac{1}{2}\mathop \sum \limits_{t = 1}^{T} \left\{ {n\ln \left( {2\pi } \right) + ln\left| {D_{t} } \right|^{2} + ln\left| {R_{t} } \right| + \varepsilon_{t}^{^{\prime}} D_{t}^{ - 2} \varepsilon_{t} } \right\}$$

In addition to the Gaussian distribution of the DCC-GARCH model, Pesaran and Pesaran (2007) also suggest the *t*-DCC-GARCH model that uses the devolatized digital asset returns represented as ${r}_{i,t-1}={r}_{it}/{\sigma }_{i,t-1}^{realized}$. The conditional correlation parameters can be estimated using the GARCH (1,1) model for conditional volatility ${\sigma }_{i,t-1}^{2}$ represented in Eq. (10):

10 $$V\left({r}_{it}|{\Omega }_{t-1}\right)= {\sigma }_{i,t-1}^{2}=\overline{{\tilde{\sigma } }_{i}^{2}}\left(1-{\lambda }_{1i}-{\lambda }_{2i}\right)+{\lambda }_{1i}{\sigma }_{i,t-2}^{2}+{\lambda }_{2i}{r}_{i,t-1}^{2}$$

where ${\tilde{\sigma }}_{i}^{2}$ is the unconditional variance of the digital asset returns. ${\lambda }_{1i}$ and ${\lambda }_{2i}$ represent the volatility parameters specific to digital assets. $\left(1-{\lambda }_{1i}-{\lambda }_{2i}\right)$ is the restriction for testing the validity of the mean-reverting feature of volatility. If the term $\left(1-{\lambda }_{1i}-{\lambda }_{2i}\right)$ is equal to zero, the estimations indicate that the model exhibits an integrated GARCH (IGARCH) process.

Finally, the empirical specification is based on multiscale correlation techniques using wavelet power spectrum, wavelet coherence, and wavelet cross-spectrum analyses. The wavelet models have a technical advantage for examining the relationship among various digital assets, both at different time horizons and frequency bands. Therefore, these models consider investors’ operations at different time scales. They capture the low- and high-scale effects of financial shocks that may occur within and across cryptocurrency markets. Theoretically, the wavelet approach is used to decompose the time series into different frequency components without confronting a loss in the time dimension. Therefore, the implementation of wavelet methods enables us to capture volatility spillovers in cryptocurrency markets for different time scales. A wavelet (i.e., ${\psi }_{u,s}(t)$) can be denoted as a real-valued square-integrable function:

11 $${\psi }_{u,s}\left(t\right)=\psi \frac{(t-u)}{\sqrt{s}}$$

where $u$ represents the location, $s$ is the scale (including both low- and high scales), and $t$ is the time dimension. One of the major characteristics of wavelet formation is the zero-mean assumption, i.e., ${\int }_{-\infty }^{\infty }\psi \left(t\right)dt=0.$ However, it is usually normalized to one such that ${\int }_{0}^{\infty }{\psi }^{2}\left(t\right)dt=1$. Thus, a time series can be transformed into wavelet formation if the following condition is satisfied:

12 $${C}_{\psi }\left(t\right)={\int }_{0}^{\infty }\frac{{\left|\psi (f)\right|}^{2}}{f}df<+\infty$$

where $\psi (f)$ denotes the Fourier transform of a given wavelet. The Morlet wavelet transformation is used to construct the wavelet power spectrum by employing Eq. (13).

13 $$\psi \left(t\right)={\pi }^{-\frac{1}{4}}{e}^{-i{\omega }_{0}t}{e}^{\frac{-{t}^{2}}{4}}$$

where ${\omega }_{0}$ denotes the central frequency. The wavelet transformation of time series $x(t)$ and $y(t)$ can be estimated by Eqs. (14) and (15), respectively, as follows:

14 $${W}_{x}\left(\tau ,s\right)={\int }_{-\infty }^{\infty }x\left(t\right)\frac{1}{\sqrt{\left|s\right|}}{\psi }^{*}\left(\frac{t-\tau }{s}\right)dt$$

15 $${W}_{y}\left(\tau ,s\right)={\int }_{-\infty }^{\infty }y\left(t\right)\frac{1}{\sqrt{\left|s\right|}}{\psi }^{*}\left(\frac{t-\tau }{s}\right)dt$$

Considering the transformation for time series $x(t)$ and $y(t)$ above, the wavelet power spectrum (WPS) can be designed to estimate the extent of volatility across various time dimensions:

16 $${WPS}_{x(y)}={\left|{W}_{x\left(y\right)}(\tau ,s)\right|}^{2}$$

The WPS can be extended to a bivariate level using two analyses: wavelet coherence and wavelet cross-spectrum. The wavelet coherence between two-time series $x(t)$ and $y(t)$ can be denoted as follows:

17 $${R}_{x,y}^{2}\left(\tau ,s\right)=\frac{{\left|S({s}^{-1}{W}_{{x}_{i}{x}_{j}}\left(\tau ,s\right))\right|}^{2}}{S\left({s}^{-1}{\left|{W}_{{x}_{i}}\left(\tau ,s\right)\right|}^{2}\right)*S\left({s}^{-1}{\left|{W}_{{x}_{j}}\left(\tau ,s\right)\right|}^{2}\right)}$$

where ${R}^{2}\epsilon [\mathrm{0,1}]$. $S$ is the smooth operator, $\tau$ is the location parameter, $s$ is the scale parameter, ${W}_{{x}_{i}{x}_{j}}$ is the wavelet cross-spectrum between $x$ and $y$, and ${W}_{{x}_{i}}$ and ${W}_{{y}_{j}}$ are the wavelet transformations of $x$ and $y$, respectively. The core reason for employing wavelet coherence is to estimate the extent of the correlation between two series across different frequencies without confronting any information loss. The Monte Carlo method was also used to measure the statistical significance of the wavelet coherence. The wavelet cross-spectrum for the series $x$ and $y$ at time *t* can be represented as follows:

18 $${C}_{x,y}\left(a,b\right)=X*\left(a,b\right)Y(a,b)$$

where $X\left(a,b\right)$ and $Y\left(a,b\right)$ are the wavelet coefficients of $x(t)$ and $y(t)$, and * denotes the complex conjugate. According to Eq. (18), the wavelet cross-spectrum is represented at each point in the time horizon. ${C}_{x,y}$ is supposed to be complex, and thus, the absolute value is estimated by:

19 $${\left|{C}_{x,y}\left(a,b\right)\right|}^{2}={\left|X*\left(a,b\right)Y(a,b)\right|}^{2}={\left|X(a,b)\right|}^{2}{\left|Y(a,b)\right|}^{2}$$

which is used for visualization. The main reason for implementing the wavelet cross-spectrum is to examine how the variance in one series affects the other across different time frequencies.

## Results and discussion

First, the descriptive statistics are presented in Table 1 to acquire summary information for the selected cryptocurrencies during the COVID-19 pandemic. The minimum and maximum values show that the prices of those assets are not stable across different time scales, which refers to the initial question of volatile behavior in cryptocurrency markets. In particular, the closing prices of these seven cryptocurrencies—except the USDT since it is pegged to the US dollar—are positively skewed throughout time, and their distribution of returns is not unique, suggesting that they are leptokurtic and mesokurtic. Therefore, this period is characterized as having an extreme increase in the prices of digital assets, thereby indicating the potential for the emergence of volatility spillovers across cryptocurrency markets.

**Table 1:** Descriptive statistics

	BTC	ETH	XLM	XRP	USDT	ADA	LTC	EOS
Mean price ($)	12,056	337	0.089	0.255	1.001	0.093	60.21	2.885
Median price ($)	9638	240	0.073	0.234	1.001	0.083	48.67	2.699
Maximum price ($)	40,519	1411	0.315	0.684	1.021	0.393	175.6	5.371
Minimum price ($)	4955	108	0.034	0.139	0.978	0.024	32.05	1.856
Total return (%)	717.7	1206	826.5	392.1	4.39	15.37	447.9	189.4
Cumulative returns (%)	281.2	651.7	284.1	4.68	0.25	703.2	139.9	− 19.9
Standard deviation	6973	247	0.055	0.097	0.002	0.067	27.63	0.566
Skewness	2.317	2.261	2.410	2.567	− 1.380	2.014	2.265	1.844
Kurtosis	8.046	8.528	8.932	9.501	51.46	8.112	7.862	6.879
Jarque–Bera	852.7	926.5	1061.4	1246.5	42,805.3	769.5	802.1	520.6
Probability	0.000	0.000	0.000	0.000	0.000	0.000	0.000	0.000
Observations	436	436	436	436	436	436	436	436

The abbreviations of the series can be listed as follows: *BTC*: Bitcoin, *ETH*: Ethereum, *XLM*: Stellar, *XRP*: Ripple, *USDT*: Tether, *ADA*: Cardano, *LTC*: Litecoin, *EOS*: Eos. All the data is obtained from the CoinDesk, covering the period from November 17, 2019 until January 25, 2021. The total return is measured based on the maximum and minimum prices of crypto assets. The cumulative returns are calculated yearly

The obtained values show that the series are highly volatile. The average return of BTC ($12,056) is the highest among the selected digital assets. The average return of XLM (0.089) is the lowest return relative to the others. The standard deviation shows the risk or volatility of returns in cryptocurrency markets. The standard deviation of the BTC market returns is 6973, which is the highest, whereas the standard deviation of the USDT market returns is 0.002, which is the lowest among the selected cryptocurrencies. The skewness of seven out of eight of the return series is positive, which indicates that the overall performance of cryptocurrency markets is favorable and symmetric in a given return series; it is highly correlated with the overall behavior of investors during the COVID-19 outbreak. The kurtosis values are above 3, which means that the data are not normally distributed (Balanda and MacGillivray 1988). In addition, the Jarque–Bera normality test shows that the null hypothesis of normality is rejected at the 1% significance level.

**Fig. 1:**
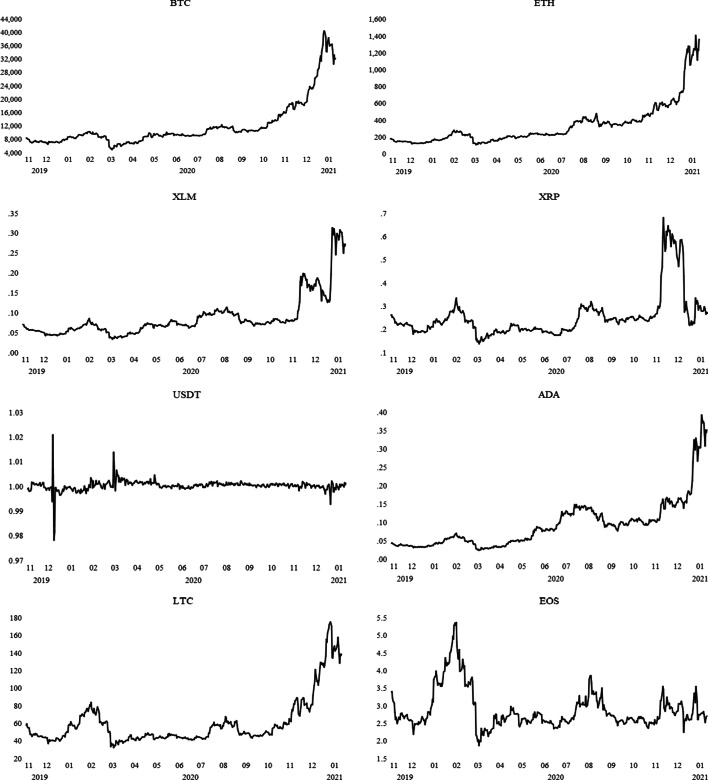
Dynamics of daily cryptocurrency prices. *Source*: CoinDesk

The time-series dynamics are shown in Fig. 1, which indicates that the prices of cryptocurrencies exhibited a change in mean and variance during the COVID-19 pandemic. In particular, five cryptocurrency prices (i.e., BTC, ETH, XLM, ADA, and LTC) showed simultaneous increases starting from September 2020 in response to a second phase of lockdowns across the globe. In particular, the prices of BTC and ETH sharply increased to approximately $40,000, and $1400, respectively, and thus, two of them trend observably higher relative to the others. A similar dynamic of a rise in the demand for such cryptocurrencies indicates a high level of correlation among the series.

Related to the *potential* channels—that is, explosive behavior and herding behavior—that drive shift to the cryptocurrency market, this also led us to understand why only three of the selected cryptocurrencies, Bitcoin, Ethereum, and Litecoin, behaved differently and were exposed to volatility spillover. As the facts of each asset are illustrated in Fig. 1, there is a relatively high demand for Bitcoin, Ethereum, and Litecoin, where their prices have an upward swing during the sample period. In this sense, the critical assumption of this study is that an excess demand for such assets can result in a volatility spillover in crypto markets. To compare and contrast demand behavior through the price mechanism, this study also considers other digital assets that are less likely to experience an upward trend in their prices. The empirical findings show that increased demand for Bitcoin, Ethereum, and Litecoin coincides with an increase in volatility. This result leads us to posit that volatility transmission should be considered together with the price dynamics of assets. In the existing literature, most studies focus on specific digital assets, mostly on Bitcoin, and therefore fall short of comparing the rest. However, this situation contains potential factors that might result in an incorrect generalization of the volatility spillover in the cryptocurrency market. In the current study, the main aim is to show that the movements in prices of those assets might have the potential to affect the buying behavior of financial investors to invest in such assets, in which their prices have an upward trend over time. Thus, they may stimulate a price bubble along with an increase in volatility spillover. Therefore, rather than focusing on specific cryptocurrencies, this study also considers relatively stable prices during the COVID-19 pandemic.

**Fig. 2:**
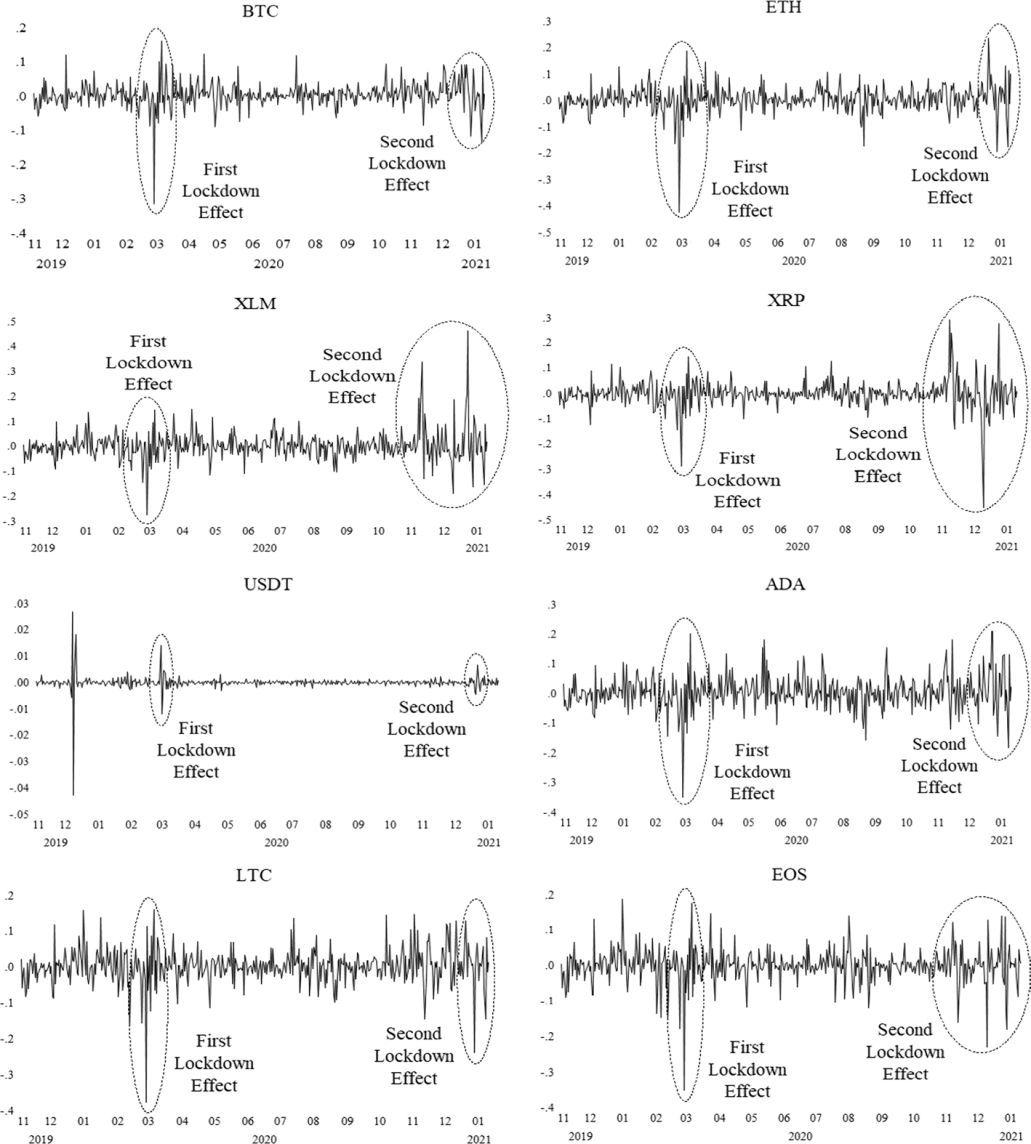
Dynamics of daily cryptocurrency market returns. *Source*: CoinDesk, Authors’ calculation

Furthermore, the dynamics of daily cryptocurrency market returns are shown in Fig. 2, and the series exhibits mean-reverting behavior with volatility clustering. The return volatility of the USDT during the COVID-19 pandemic is low, whereas the others are relatively high. Hence, the estimates of preliminary tests suggest that the DCC-GARCH model is relevant for capturing the volatility spillovers in the cryptocurrency market during the COVID-19 outbreak.

Implementing GARCH family models requires meeting various preliminary stationary and diagnostic tests such as the unit-root test, Ljung–Box Q-statistics, Lagrange multiplier (LM) test, and ARCH effect. The results are shown in Table 2. To justify whether the series has a unit root, the ADF tests of selected cryptocurrency prices show that the series are non-stationary at level, but stationary in their first differences. The normality tests imply that all series are not normally distributed in Jarque–Bera statistics. Meanwhile, the Ljung-Box Q-statistics show that the serial correlation among the series is statistically significant. The null hypothesis of no serial correlation is rejected at the 1% significance level for all the series. In addition, the results of the LM test also showed the same pattern as the results of the Q-statistics test. Furthermore, the ARCH test points to the case in which the series have no constant variance, indicating an ARCH effect for all series.

**Table 2:** Stationary and residual diagnostic tests

	BTC	ETH	XLM	XRP	USDT	ADA	LTC	EOS
ADF (Level)	− 1.676	2.660	0.791	− 3.384	− 3.329	0.605	− 0.751	− 2.872
	(0.760)	(1.000)	(0.999)	(0.055)	(0.063)	(0.999)	(0.967)	(0.173)
ADF (1st difference)	− 4.692	− 5.071	− 6.613	− 13.87	− 14.39	− 4.093	− 6.551	− 21.99
	(0.001)	(0.001)	(0.000)	(0.000)	(0.000)	(0.007)	(0.000)	(0.000)
Q-statistics	7696.2	2949.4	5826.2	5171.9	329.8	6590.0	6785.8	4761.3
	(0.000)	(0.000)	(0.000)	(0.000)	(0.073)	(0.000)	(0.000)	(0.000)
Normality test	852.8	926.5	1061.4	1246.5	42,805.3	769.5	802.0	520.6
	(0.000)	(0.000)	(0.000)	(0.000)	(0.000)	(0.000)	(0.000)	(0.000)
LM test	1625.4	1208.7	615.5	379.9	3.059	818.8	660.3	160.1
	(0.000)	(0.000)	(0.000)	(0.000)	(0.000)	(0.000)	(0.000)	(0.000)
ARCH effect	1096.1	955.4	285.2	136.5	27.27	751.4	540.1	102.8
	(0.000)	(0.000)	(0.000)	(0.000)	(0.000)	(0.000)	(0.000)	(0.000)

*p*-values are given in parentheses. For the Augmented Dickey-Fuller (ADF) test, the trend and intercept are included in the test equation. The lag length is selected through the Akaike Information Criterion (AIC). The lag length for the Q-statistics is selected as 36. The normality test statistics reflect the Jarque–Bera test statistics. The null hypothesis in the LM test is no serial correlation at up to 36 lags. The ARCH effect is selected to detect heteroskedasticity among the series, which regresses the squared residuals on lagged squared residuals and a constant at up to 36 lags

Further, Table 3 describes more detailed estimation findings for major residual diagnostic tests (i.e., Ljung-Box Q and Q^2^ statistics and ARCH-LM statistics) along with different lag structures. The results show that all the series have ARCH effects. In addition, the series have a serial correlation in the case of both Q and Q^2^ statistics. Therefore, the residual diagnostic tests reveal that the GARCH family models can provide statistically reliable results compared to traditional ARCH models, which leads us to test the EGARCH and DCC-GARCH models empirically.

**Table 3:** Ljung–Box and ARCH-LM tests

	Q (10)	Q (20)	Q^2^ (10)	Q^2^ (20)	ARCH-LM(5)	ARCH-LM(10)	ARCH-LM(20)
BTC	3705.9 (0.000)	5975.6 (0.000)	3180.8 (0.000)	4189.5 (0.000)	2890.4 (0.000)	1461.9 (0.000)	1543.5 (0.000)
ETH	3397.4 (0.000)	5361.6 (0.000)	2486.7 (0.000)	3149.0 (0.000)	1513.5 (0.000)	1026.5 (0.000)	1377.1 (0.000)
XLM	3133.9 (0.000)	4522.3 (0.000)	2300.5 (0.000)	2628.9 (0.000)	978.8 (0.000)	504.3 (0.000)	343.4 (0.000)
XRP	3298.1 (0.000)	4750.5 (0.000)	2642.1 (0.000)	3385.8 (0.000)	948.1 (0.000)	475.7 (0.000)	244.3 (0.000)
USDT	182.2 (0.000)	255.5 (0.000)	239.3 (0.000)	239.8 (0.000)	104.9 (0.000)	52.2 (0.000)	24.9 (0.000)
ADA	3296.0 (0.000)	5072.0 (0.000)	2225.7 (0.000)	2600.9 (0.000)	1298.3 (0.000)	998.3 (0.000)	1129.7 (0.000)
LTC	3543.8 (0.000)	5656.3 (0.000)	2900.5 (0.000)	4043.7 (0.000)	1435.9 (0.000)	825.7 (0.000)	688.9 (0.000)
EOS	3040.1 (0.000)	4457.7 (0.000)	2346.9 (0.000)	2914.6 (0.000)	735.8 (0.000)	400.4 (0.000)	194.3 (0.000)

*p*-values are given in parentheses

Table 4 presents the findings of the correlation matrix of the prices of the selected cryptocurrencies. The results comprise both positive and negative values, which shows that cryptocurrency markets do not even slightly move in the same direction. Furthermore, the correlation among the prices of cryptocurrencies ranges from 0.007 to 0.972, which indicates that some values are higher than 0.80, suggesting high co-movements and multicollinearity among the series. The correlation between ADA and EOS prices was the lowest, while the correlation between BTC and ETH was the highest at their absolute values. This indicates that the correlation between the COVID-19 pandemic and the most popular cryptocurrencies is higher, while the correlation between the least popular cryptocurrencies is lower. However, Pearson product-moment-based unweighted ordinary results of correlation values provide an average correlation without handling the variations in the corrections. Therefore, a more detailed correlation was obtained using the DCC-GARCH model and wavelet analysis.

**Table 4:** Correlation matrix

	BTC	ETH	XLM	XRP	USDT	ADA	LTC	EOS
BTC	1.000							
ETH	0.972	1.000						
XLM	0.926	0.943	1.000					
XRP	0.471	0.476	0.582	1.000				
USDT	− 0.091	− 0.036	− 0.032	− 0.026	1.000			
ADA	0.911	0.958	0.946	0.441	− 0.020	1.000		
LTC	0.948	0.905	0.869	0.468	− 0.110	0.824	1.000	
EOS	0.026	0.021	0.077	0.287	− 0.081	0.007	0.271	1.000

The parameter estimation results of the EGARCH (1,1,1) model are presented in Table 5. One of the main advantages of using the EGARCH model is that it considers the logarithm of volatility. The ADF unit root test statistics show that the series were not stationary at the 1% significance level, and the EGARCH model provided flexibility to get reliable and robust estimation results. Further, unlike the GARCH model, the use of EGARCH model depends on the fact that it allows for no restriction of alpha and beta in the estimation procedure to be larger than zero (Chang and McAleer 2017; Martinet and McAleer 2018). In other words, the EGARCH model estimations are not restricted compared to the other models in the GARCH family. The specification of the conditional variance equation in the EGARCH model also distinguishes this model from the other models.

**Table 5:** The estimation results of EGARCH (1,1,1) model

	BTC	ETH	XLM	XRP	USDT	ADA	LTC	EOS
Method: ML ARCH – Normal Distribution (OPG—BHHH / Line Search Steps)								
$$Mean Equation:d{X}_{t}=\mu +{\o }_{1}d{X}_{t-1}+{\o }_{2}d{X}_{t-2}+{\theta }_{1}{u}_{t-1}+{\theta }_{2}d{u}_{t-2}+{u}_{t}$$								
$\mu$	36.84 (0.039)	1.186 (0.023)	0.001 (0.240)	0.001 (0.704)	− 2.621 (0.203)	0.000 (0.052)	0.169 (0.177)	0.001 (0.000)
${\o }_{1}$	1.816 (0.000)	1.282 (0.000)	1.972 (0.000)	− 1.096 (0.000)	1.078 (0.000)	1.559 (0.000)	1.688 (0.000)	1.124 (0.000)
${\o }_{2}$	− 0.913 (0.000)	− 0.947 (0.000)	− 0.975 (0.000)	− 0.945 (0.000)	− 0.353 (0.000)	− 0.921 (0.000)	− 0.939 (0.000)	− 0.164 (0.000)
${\theta }_{1}$	− 1.843 (0.000)	− 1.272 (0.000)	− 1.969 (0.000)	1.136 (0.000)	− 1.699 (0.000)	− 1.592 (0.000)	− 1.691 (0.000)	− 1.276 (0.000)
${\theta }_{2}$	0.933 (0.000)	0.976 (0.000)	0.971 (0.000)	0.992 (0.000)	0.832 (0.000)	0.979 (0.000)	0.947 (0.000)	0.283 (0.000)
$$Variance Equation={\mathrm{log}(\sigma }_{t}^{2})=\omega +\sum_{j=1}^{q}{\eta }_{j}\mathrm{log}\left({\sigma }_{t-j}^{2}\right)+\sum_{i=1}^{p}{\gamma }_{i}\left|\frac{{\varepsilon }_{t-i}}{{\sigma }_{t-i}}\right|+\sum_{k=1}^{r}{\lambda }_{k}\frac{{\varepsilon }_{t-k}}{{\sigma }_{t-k}}$$								
$\omega$	− 0.030 (0.581)	− 0.090 (0.000)	− 0.676 (0.000)	− 0.761 (0.000)	− 2.067 (0.000)	− 0.222 (0.000)	− 0.077 (0.000)	− 0.013 (0.188)
${\eta }_{j}$	0.113 (0.000)	0.174 (0.000)	0.416 (0.000)	0.495 (0.000)	0.658 (0.000)	0.187 (0.000)	0.142 (0.000)	− 0.083 (0.000)
${\gamma }_{i}$	0.043 (0.000)	0.102 (0.000)	0.136 (0.000)	0.129 (0.000)	− 0.215 (0.000)	0.108 (0.000)	0.103 (0.000)	0.156 (0.000)
${\lambda }_{k}$	0.997 (0.000)	0.996 (0.000)	0.964 (0.000)	0.954 (0.000)	0.884 (0.000)	0.991 (0.000)	0.992 (0.000)	0.981 (0.000)
${e}^{\lambda }$	1.044	1.107	1.146	1.138	0.807	1.114	1.108	1.169
$AIC$	14.72	8.022	− 8.125	− 6.195	− 10.01	− 8.085	4.837	− 1.324
$SIC$	14.81	8.107	− 8.040	− 6.110	10.93	− 8.000	4.922	− 1.239
$DW Stat.$	1.737	1.962	1.747	1.604	2.548	1.783	1.823	1.808
$Log Likelihood$	− 3179.1	− 1727.8	1768.0	1350.2	2392.8	1759.5	− 1038.3	295.6
$ARCH-LM$	0.254 (0.990)	0.654 (0.767)	1.038 (0.410)	0.591 (0.822)	0.472 (0.908)	1.237 (0.265)	0.355 (0.965)	0.976 (0.464)

*p*-values are given in parentheses. $\omega$ is the constant, $\eta$ is the ARCH effect, $\gamma$ is the asymmetric effect, and $\lambda$ is the GARCH effect. Presample variance is selected as backcast with a parameter equal to 0.7. Coefficient covariance is computed using an outer product of gradients (OPG). Error distribution is selected as Gaussian. The optimization method is OPG – Berndt-Hall-Hall-Hausman (BHHH) algorithm with a line search step. Variable *X* in the mean equation consists of selected cryptocurrencies, respectively used in estimating models. The variables are estimated in their first differences, depending on unit-root results presented in Table 2. *ø*_1_ and *ø*_2_ show AR(1) and AR(2) coefficients, respectively. *θ*_1_ and *θ*_2_ show MA(1) and MA(2) coefficients, respectively. *μ* is the white-noise disturbance term. In consideration of the ARCH – LM test, the heteroskedasticity is tested up to 10 lags

The results presented in Table 5 show that most of the coefficients are statistically significant in capturing the volatility spillover in the cryptocurrency market. First, the arch term (η) refers to the extent to which the magnitude of a shock to the variance has a significant impact on future volatility in the returns of cryptocurrencies. The significance of the coefficients for the *η*_*j*_ term implies that the size of the shock has a significant effect on the volatility of returns. The signs of the terms are positive for all cryptocurrencies, which show a positive relationship between the past variance and the current variance, implying that the larger the magnitude of the shock to the variances of series, the higher the volatilities over the markets.

Second, the leverage effect term (γ) gives us insight into how the sign of the shock impacts the future volatility of cryptocurrency returns. Except for the USDT market, the signs of the leverage effect terms are positive with statistically significant coefficients, which indicates that good news will increase volatility more than bad news of the same size. This is evidence of the leverage effect for those cryptocurrencies.

Finally, the GARCH term (λ) provides us with insight into the persistence of past volatility and how past volatility helps predict future volatility. The signs of the GARCH terms differ for each cryptocurrency. Therefore, the significant feature of the coefficients shows that past volatility has a persistent effect on future volatility and its predictions. However, the problem for the EGARCH model to capture volatility spillovers across markets is its inability to provide co-volatility, as the model cannot predict volatility for two or more time series. The assumption is that the current volatility of one series is influenced not only by its own past process but also by past processes of volatilities of other series. Therefore, the next issue is to predict multivariate GARCH models using the DCC-GARCH approach.

The time-varying correlations and volatilities are significant in identifying the gains and returns of cryptocurrencies. The DCC-GARCH model with a Gaussian distribution was used to detect these points based on the maximum likelihood values. Table 6 presents the maximum likelihood estimates of the volatility decay parameters (λ_1_ and λ_2_) and the correlation decay parameters (δ_1_ and δ_2_) of the Gaussian DCC-GARCH models for the cryptocurrency price series.

**Table 6:** Maximum Likelihood estimates of the Gaussian DCC model

Parameter	λ_1_	λ_2_	Probability	1 − (λ_1_ + λ_2_)
BTC	0.85137	0.09289	0.000	0.05574
ETH	0.86736	0.10921	0.000	0.02343
XLM	0.60571	0.22733	0.000	0.16696
XRP	0.64199	0.25755	0.000	0.10046
USDT	0.68949	0.29172	0.000	0.01879
ADA	0.76910	0.16656	0.000	0.06434
LTC	0.81803	0.14208	0.000	0.03989
EOS	0.81151	0.15512	0.000	0.03337

The decay factors 1–(δ_1_ + δ_2_) = 0.02414 where δ_1_ = 0.95349 and δ_2_ = 0.02237; Maximum Log-Likelihood = 2191.7

As shown in Table 6, the decay parameters are highly significant at the 1% level. In addition, the sum of λ_1_ and λ_2_ gives us insights for the case in which the conditional volatilities of the series are mean-reverting with gradual decay of volatility because the total values are less than 1 for each series, which indirectly indicates that univariate volatility estimations are reliable to account for only one time series.

Furthermore, the sum of the correlation decay parameters, where δ_1_ = 0.95349 and δ_2_ = 0.02237, is equal to δ_1_ + δ_2_ = 0.97586, indicating that the conditional correlations are also mean-reverting since the total value is less than 1 for each series. This means that the markets slowly equilibrate their returns, and indicates that the shocks slowly decayed. Therefore, the results of the volatility and correlation decay parameters indicate that investors in these markets are less likely to be confronted with an immediate loss as a consequence of shocks.

The next step was to represent the unconditional volatilities through the diagonal elements of the covariance matrix. The results imply that the value of unconditional volatility in all markets is far from unity, which is considered to have low volatility in returns. Table 7 summarizes the estimation values for unconditional volatility ranking, in which BTC has the highest value and the USDT has the lowest value, where the USDT market is the most stable within the selected cryptocurrencies. Even though each market is extremely different from the other in terms of volatility, they are far from the value of 1, which indicates a high volatility in returns.

**Table 7:** The rank of unconditional volatility

Rank	Abbreviations	Cryptocurrencies	Unconditional volatility
1	BTC	Bitcoin	611.2
2	ETH	Ethereum	28.08
3	LTC	Litecoin	3.913
4	EOS	Eos	0.152
5	XRP	Ripple	0.019
6	XLM	Stellar	0.009
7	ADA	Cardano	0.008
8	USDT	Tether	0.002

Table 8 represents the unconditional distribution of the market pairs to depict whether the co-movements between the markets are relevant. Since the Bitcoin market is located at the highest rank of unconditional volatility, it will be considered as a benchmark in the comparison of correlation between the markets. The unconditional volatility matrix of the pairs for BTC market returns with all the markets is relatively low. The results show that the BTC market has the highest correlation with the LTC (0.793) and ETH (0.691) markets and the lowest correlation with the USDT (0.027) market. It can be predicted that changes in emerging cryptocurrency markets such as Tether will have more influence on the Bitcoin market than changes in any popular cryptocurrency market such as Litecoin and Ethereum. It can be seen that the markets with pegged cryptocurrencies have low returns but also have a low-risk level relative to the other digital assets with high volatility rates. If the investors diversify their financial resources by buying different digital assets to compensate for any potential loss, they may have chosen two low correlated assets, such as Tether and Bitcoin, to balance their returns when the prices of a risky asset (i.e., Bitcoin) were negatively shocked. Therefore, if many investors follow that financial strategy, the *indirect* influence of risk-free assets on the risky asset may be more effective relative to the *indirect* influence between two risky assets. This also means that a low level of correlation indicates that the investors do not invest in only one cryptocurrency. Rather, they diversify their financial resources by buying different cryptocurrencies.

**Table 8:** The unconditional volatility matrix

	BTC	ETH	XLM	XRP	USDT	ADA	LTC	EOS
BTC	611.2	0.691	0.454	0.361	0.027	0.558	0.793	0.491
ETH	0.691	*28.08*	0.522	0.399	− 0.008	0.711	0.766	0.498
XLM	0.454	0.522	*0.009*	0.497	0.104	0.762	0.449	0.503
XRP	0.361	0.399	0.497	*0.019*	0.030	0.372	0.475	0.586
USDT	0.027	− 0.008	0.104	0.030	0.002	0.077	0.004	0.052
ADA	0.558	0.711	0.761	0.372	0.077	*0.008*	0.560	0.489
LTC	0.793	0.766	0.449	0.475	0.004	0.560	*3.913*	0.663
EOS	0.491	0.498	0.503	0.586	0.052	0.489	0.663	0.152

**Fig. 3:**
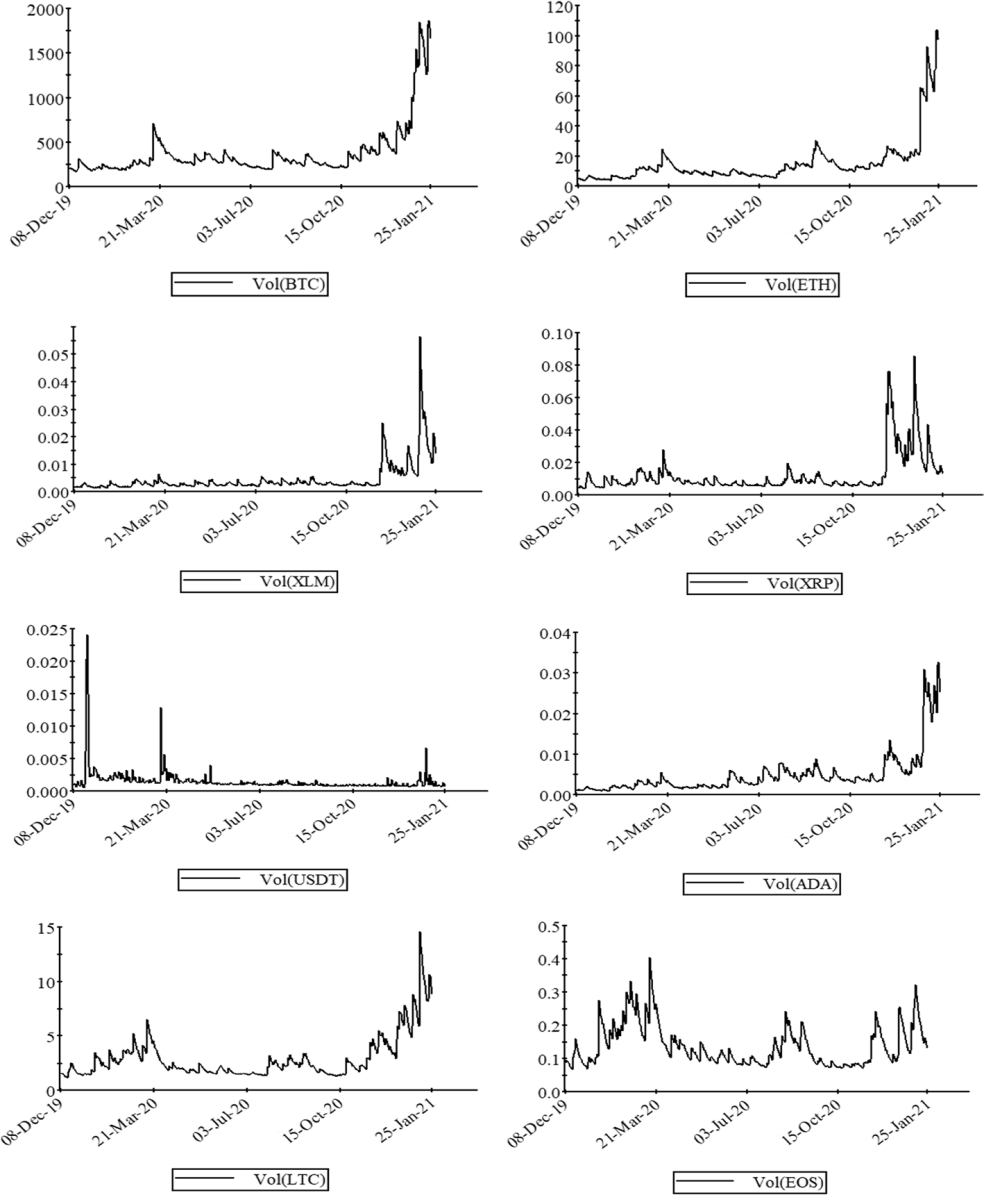
A plot of conditional volatilities of daily return

Figure 3 shows the values of conditional volatilities of cryptocurrency markets for selected assets. The values plotted on the graph imply that the volatilities have a time-varying nature. While the volatility trend is smooth for most of the cryptocurrencies, it skyrocketed after November 2020, when the second phase of the lockdown started across the globe. All markets show close movements of volatilities except USDT which acts differently from the others over the whole period. One of the major reasons for this smooth volatility in the USDT market was the herding behavior of investors toward the markets where their returns were relatively much higher. Therefore, the USDT market does not seem to be integrated with the other markets. In addition, Figures 13, 14 and 15 represent the residual, actual, and fitted values, conditional standard deviations, and conditional variations for all series in the “Appendix”, respectively.

**Fig. 4:**
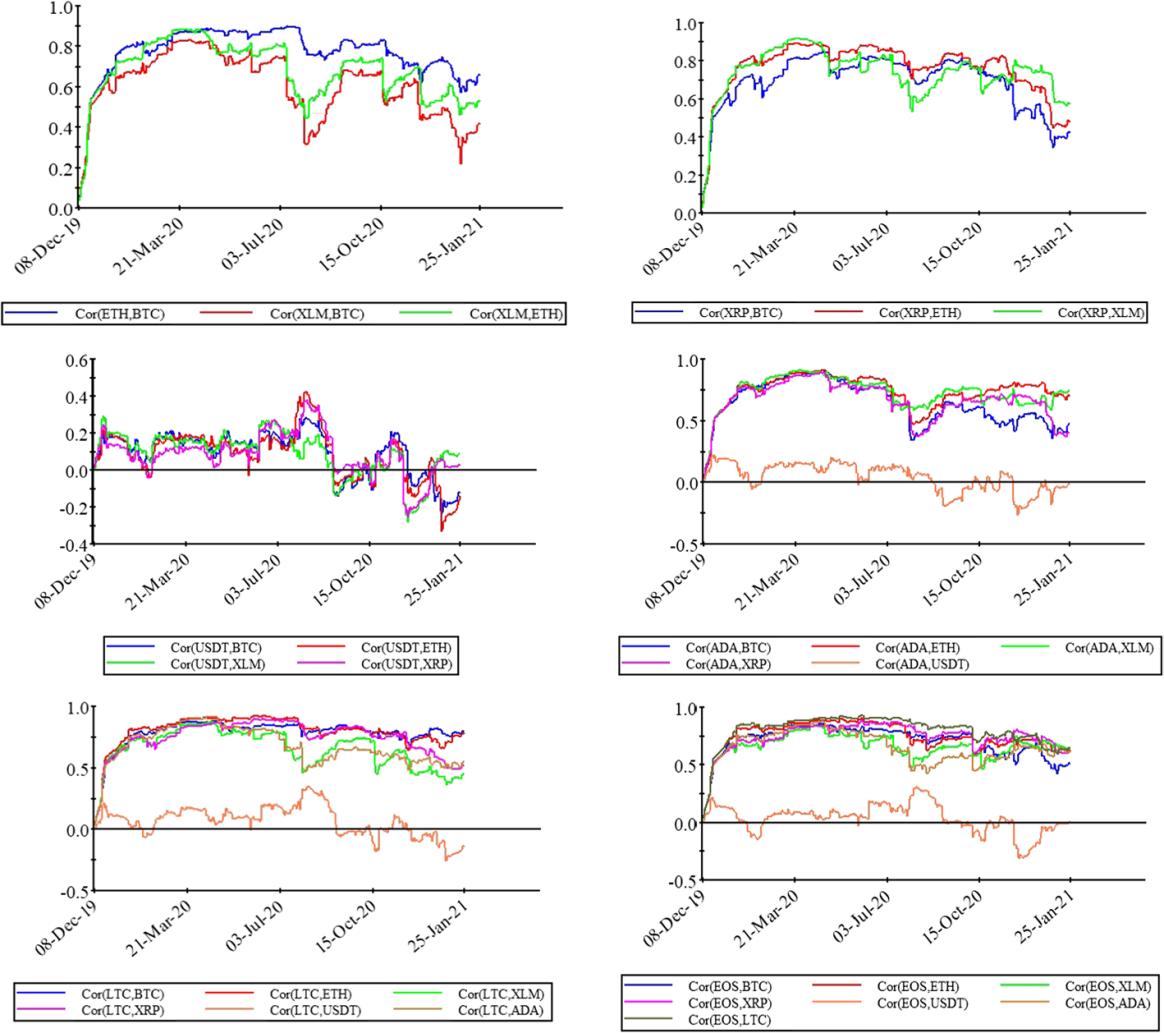
A plot of conditional correlations of selected cryptocurrencies

Figure 4 shows the conditional correlation between the cryptocurrency markets. The figure depicts the variations in the results of the conditional and unconditional correlations of cryptocurrencies. The lines representing the correlation of pairs cryptocurrency market of USDT with the other markets are mainly at the bottom crossing, with lines equal to zero showing less correlation. In other words, the returns from the USDT market show less correlation with the other selected cryptocurrency markets. The most popular cryptocurrency markets (that is BTC, ETH, LTC, and XRP) have a higher correlation with each other than the other markets. The graphical representation of these markets shows close movements of the correlation lines, suggesting that these four popular markets behave in a similar way. The COVID-19 outbreak has led to more integrated cryptocurrency markets, and has also stimulated investors’ herding behavior. As supported by the values of unconditional volatility, investors tend to buy highly demanded assets in the cryptocurrency market in parallel with a reduction in diversification benefits. However, financial markets mostly follow a cyclical and iterative pattern, indicating that investors act independently over time. In this regard, GARCH family models do not fully grasp the information of all time scales, although they control the conditional correlation and covariances. Therefore, the next step was to use various wavelet analyses to assess the changing relations that occur across different time scales, rather than the given time scale.

**Fig. 5:**
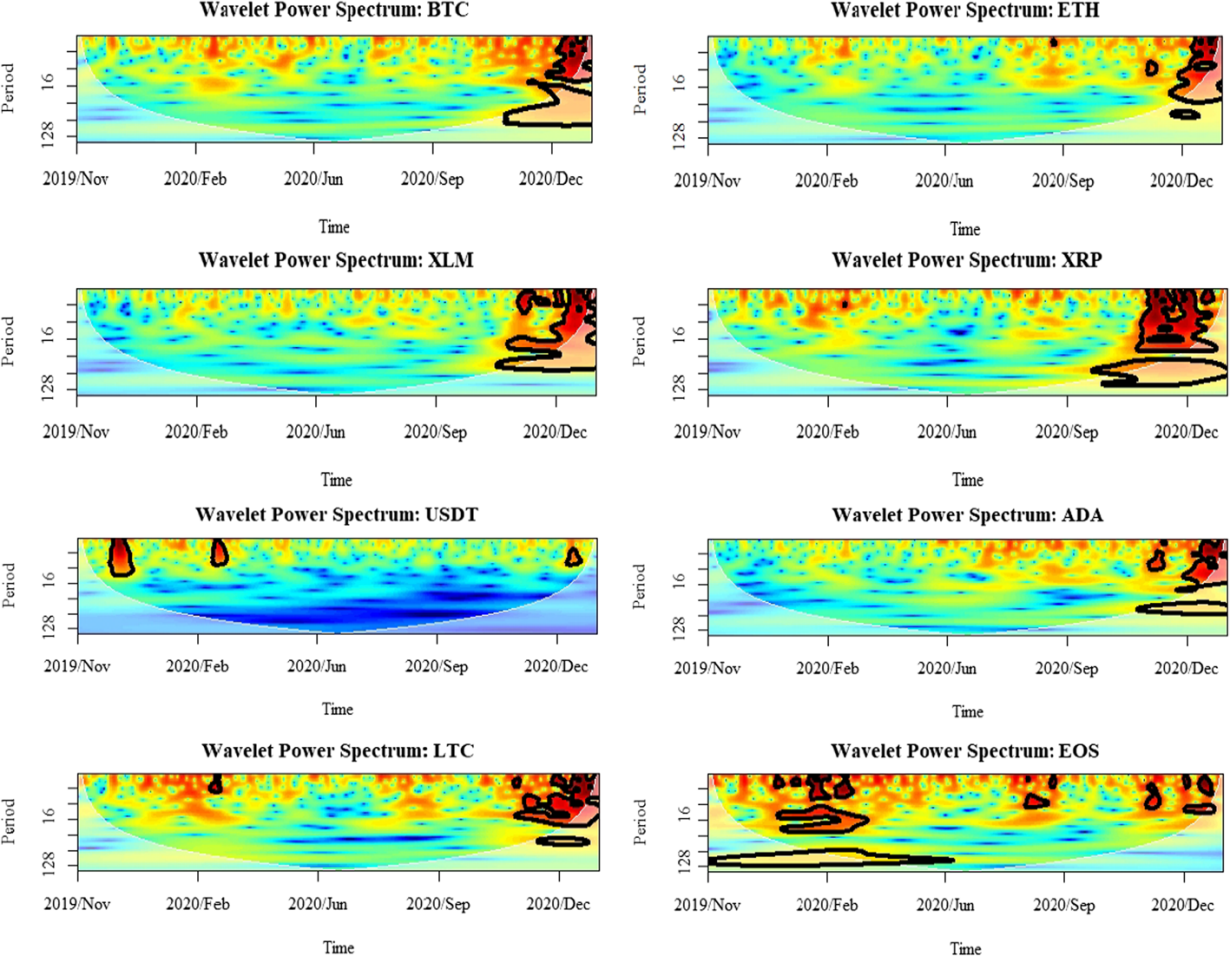
Results for wavelet power spectrum

Figure 5 shows the WPS for the selected cryptocurrency time series over the COVID-19 pandemic. The Y-axis indicates the frequency/time horizon, and the X-axis shows the time scale. The results imply that most of the power is concentrated within the last quarter of 2020 and the early phase of 2021, although there is appreciable power over longer periods. With wavelet power coherence analysis, one can see variations in the frequency of occurrence and amplitude of the COVID-19 outbreak. For instance, the global lockdowns and the slowdown of production can be implemented as one of the core factors to understand these high frequencies through the cryptocurrency markets. It also indicates that these markets were highly volatile at that time. Further, looking at the power spectrum, it is clear that the volatilities dropped from high frequency (low-scales) to low frequency (high-scales) during the second lockdown effect. This shows the market fluctuations were highly influential on financial investors of the asset investment horizons.

**Fig. 6:**
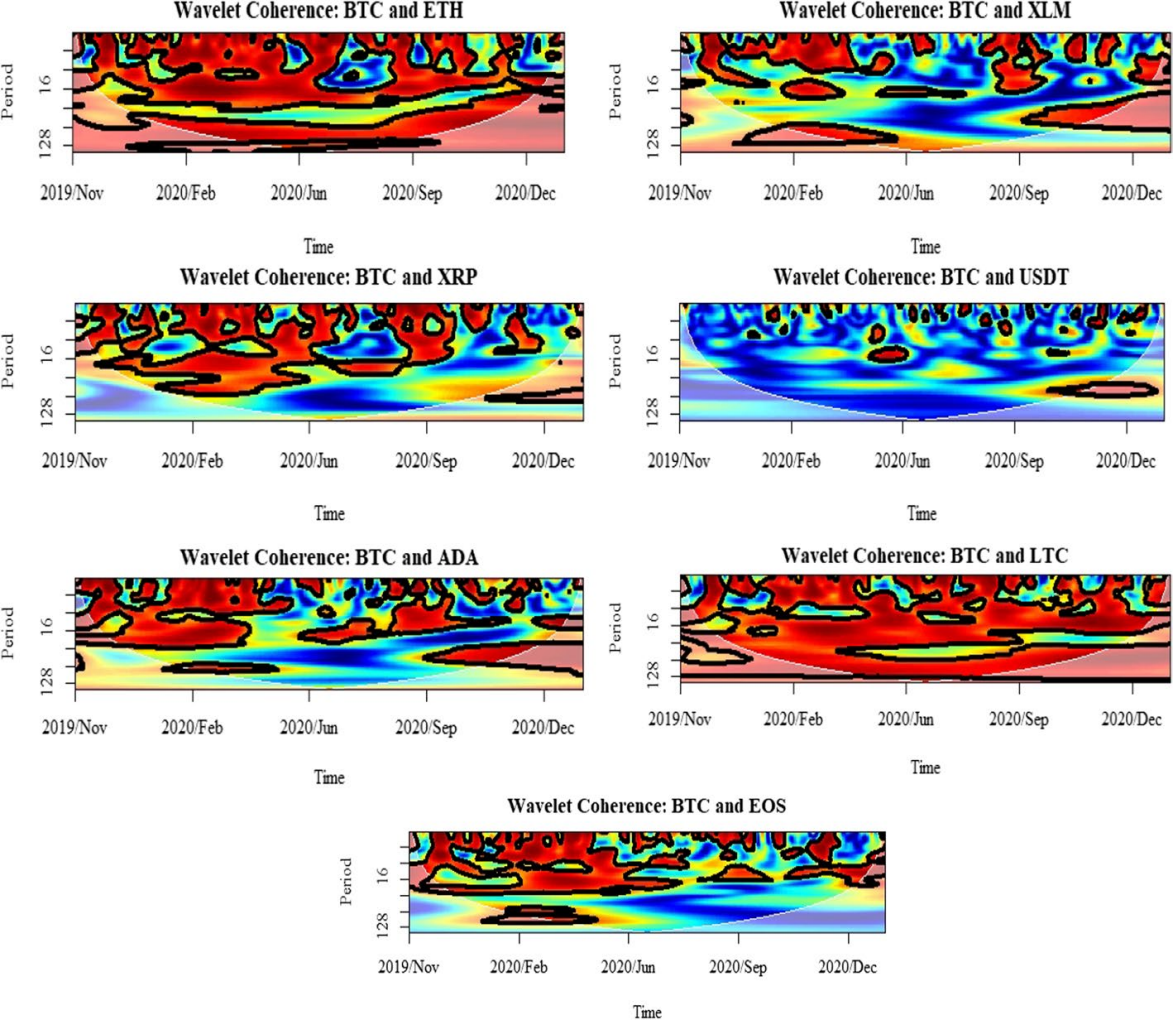
Results for wavelet coherence: BTC market

**Fig. 7:**
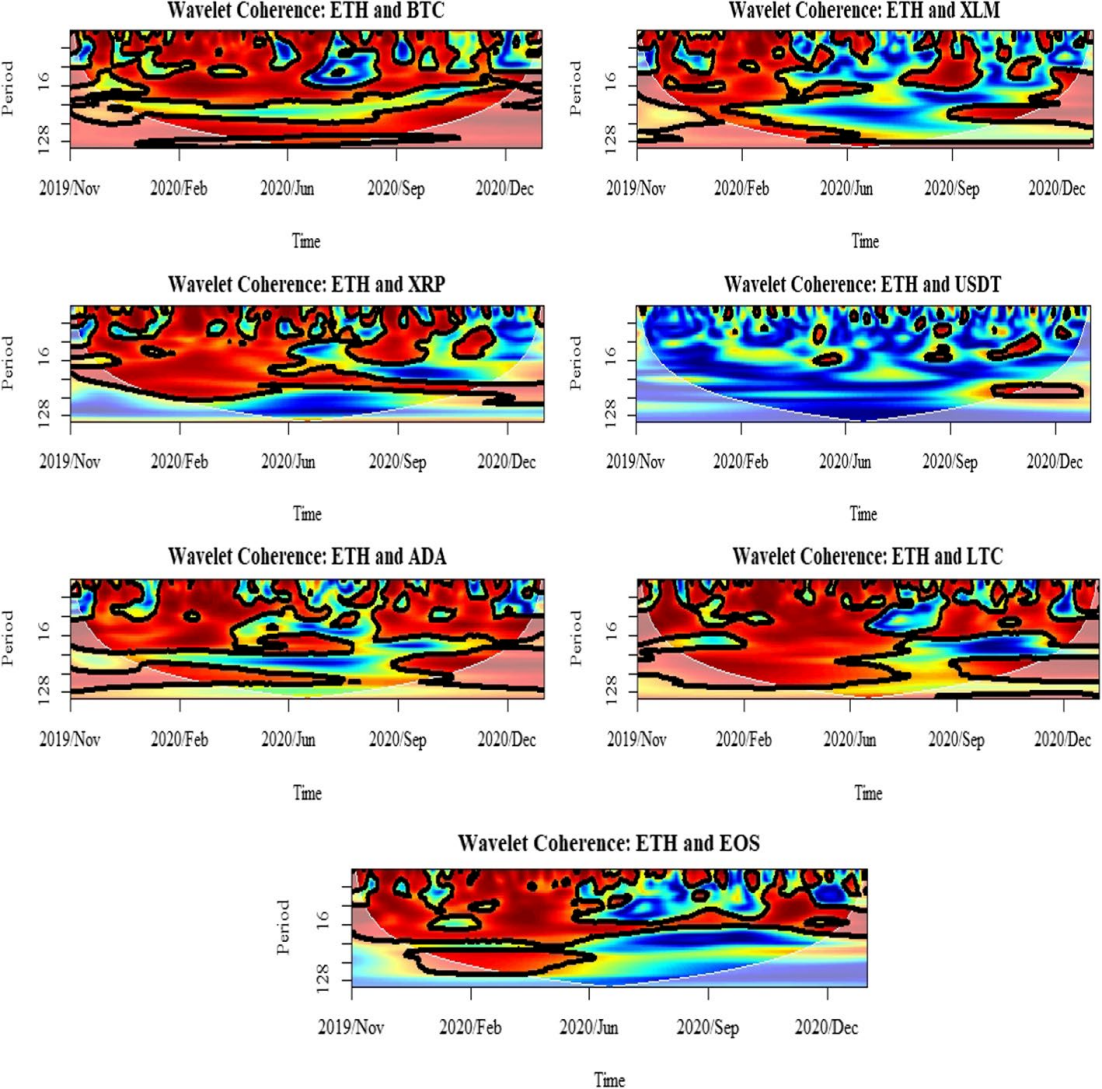
Results for wavelet coherence: ETH market

**Fig. 8:**
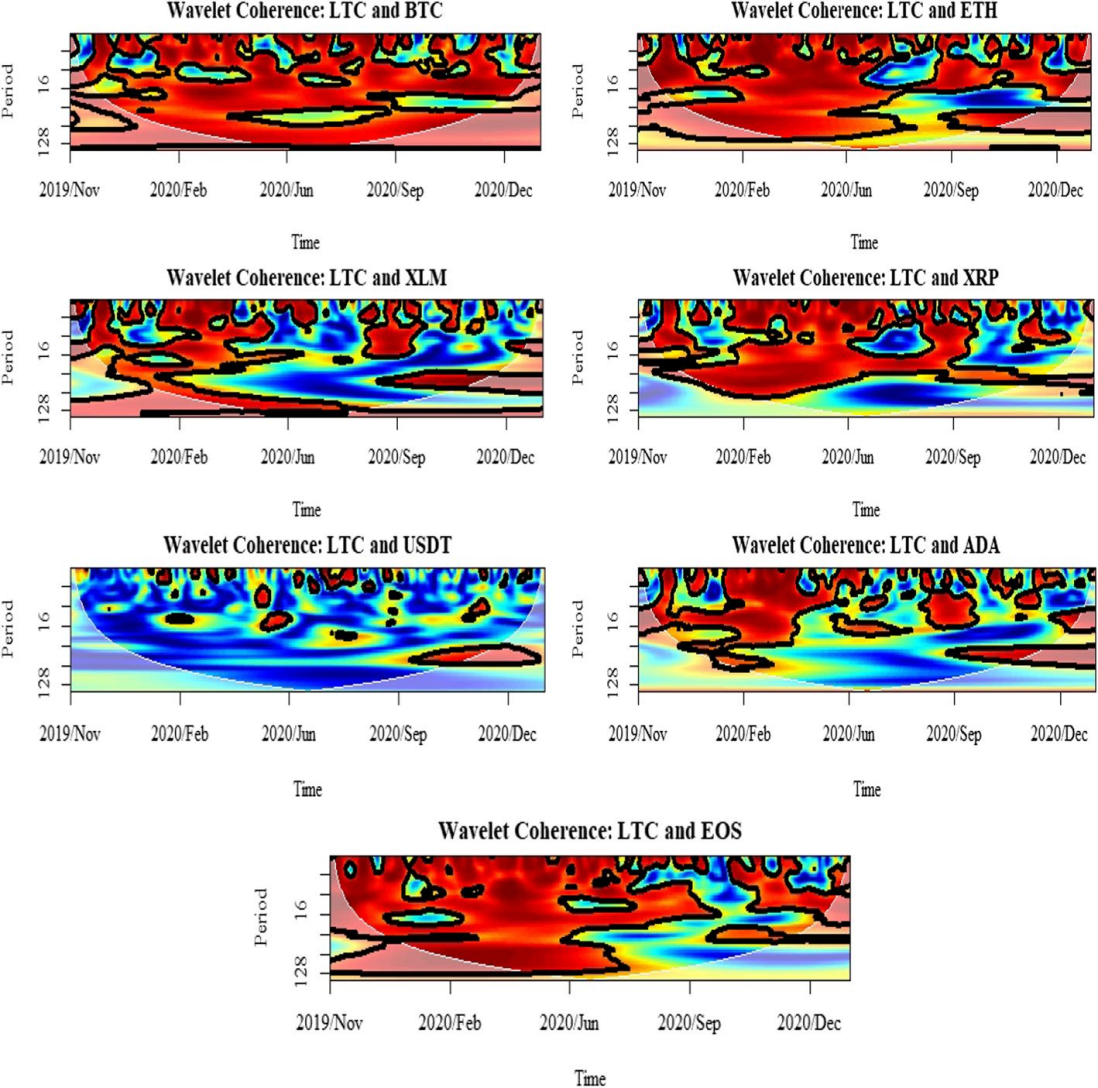
Results for wavelet coherence: LTC market

The second useful investigation was the wavelet coherence, defined as the square of the cross-spectrum, normalized by the individual power spectra. It shows how much the linear information of one asset is explained by the other, and thus, it can be used to estimate causality among the selected assets. Figures 6, 7 and 8 depict the wavelet coherence for the highest three cryptocurrencies (BTC, ETH, and LTC) in terms of their rank of unconditional volatility, given in Table 7. Also, the outputs of the conditional correlation graph in Fig. 4, show that there is a negative correlation between those markets during the periods of market turbulence which started with the COVID-19 outbreak. While the evidence from the wavelet coherence implies that financial investors were overly sensitive to the possible market crashes emerging in the COVID-19 pandemic, the investors’ behavior was more visible among those cryptocurrencies through the shifts in their preferences. The evidence of decoupling between BTC, ETH, and LTC is reflected in a decrease in conditional correlation values. From the wavelet coherence plot, it is confirmed that the initial correlations among the three assets exist between 16 and 32 days, showing that there is a significant co-movement between short-term investors. However, as the cryptocurrency markets mature, the return co-movement becomes intense. In particular, the increase in depth of return co-movement became permanent from just after the first lockdown of March 2020 up to the present with a significant coherence up to 128 days, implying a possible increased scale of long-term investors.

**Fig. 9:**
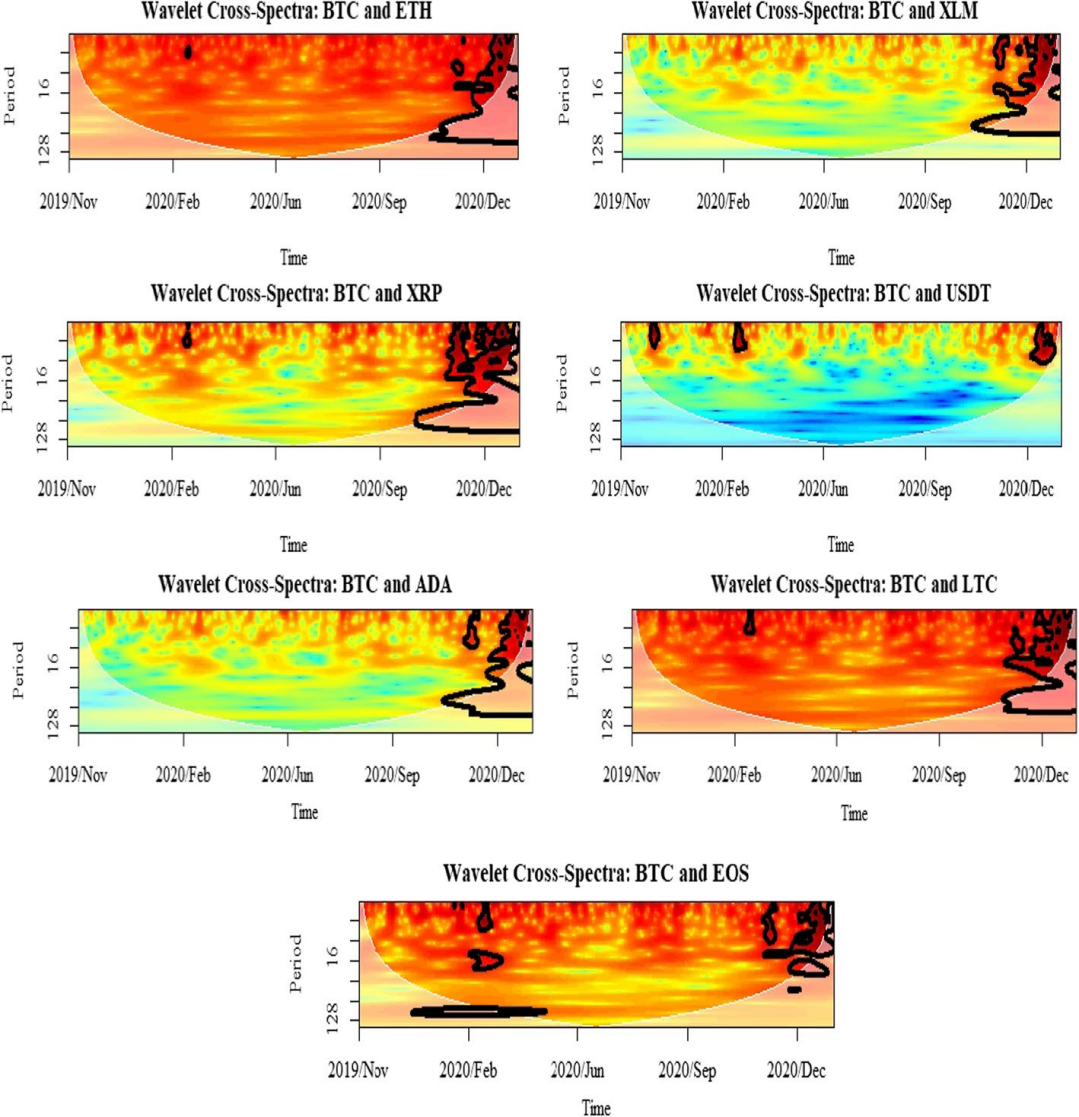
Results for wavelet cross-spectra: BTC market

**Fig. 10:**
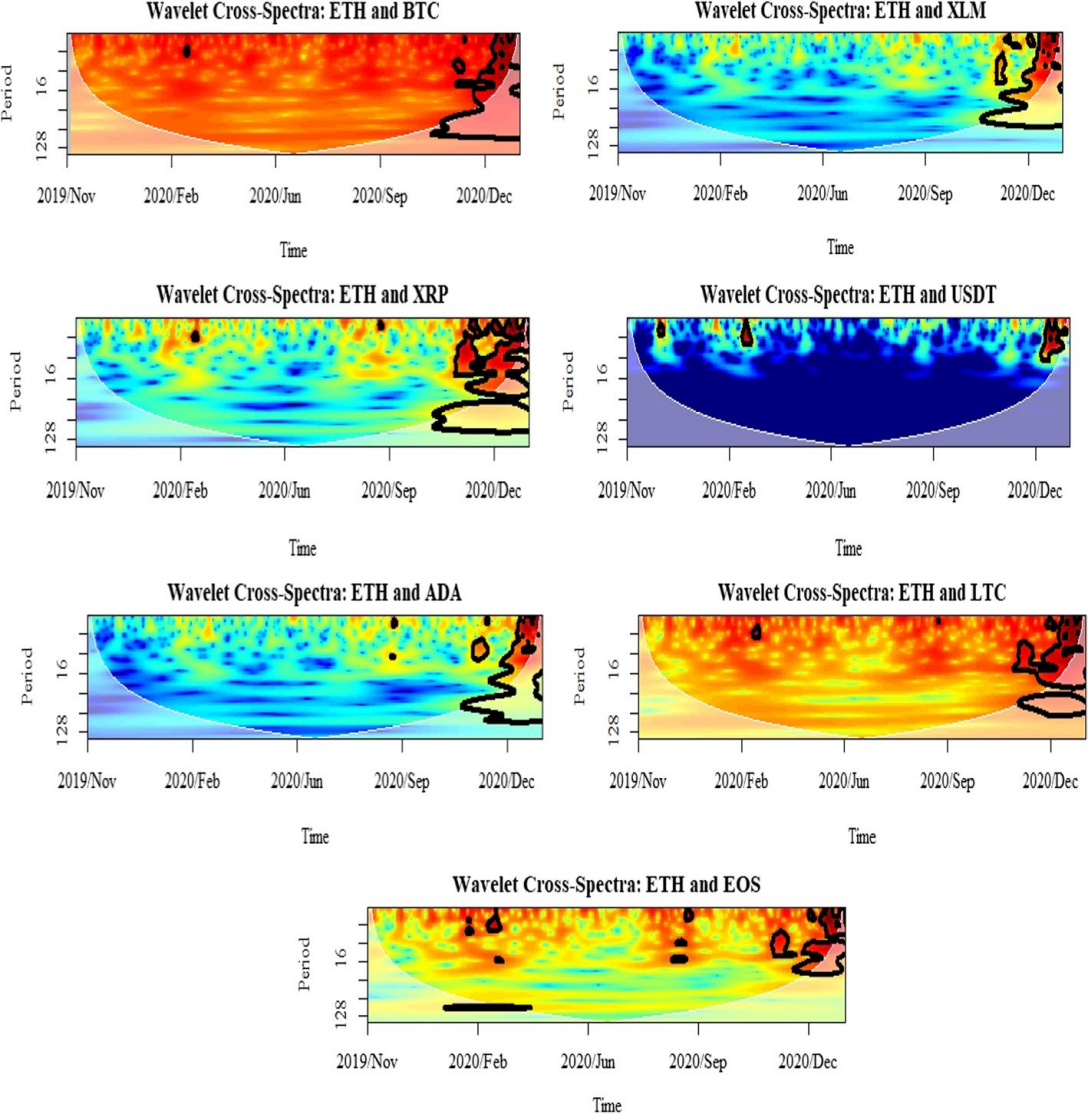
Results for wavelet cross-spectra: ETH market

**Fig. 11:**
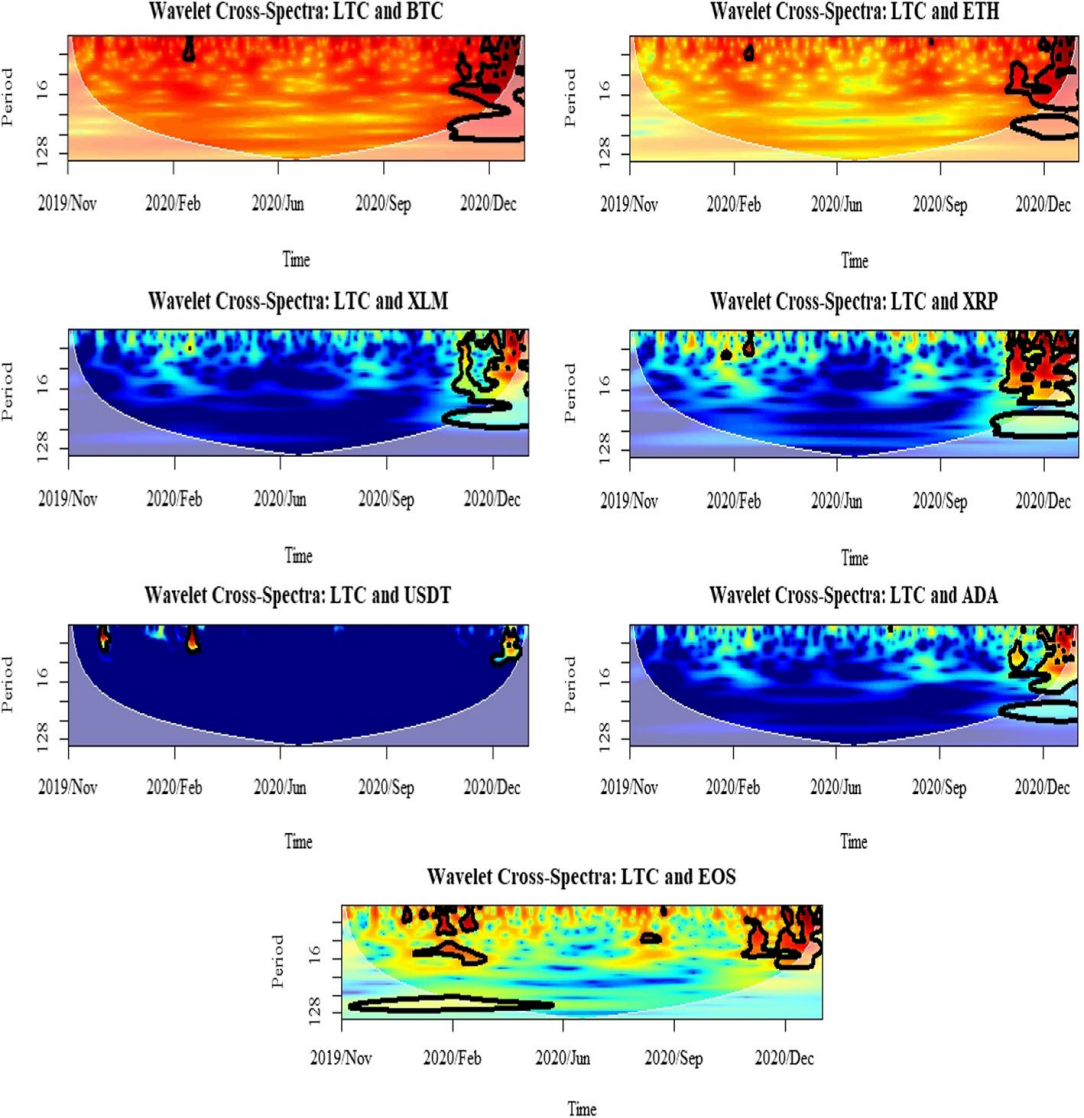
Results for wavelet cross-spectra: LTC market

Furthermore, this study uses cross-spectrum analysis to investigate the nature of volatility spillover across different time horizons. In particular, it allows for an estimation of the coherence between different assets, indicating how much linear information is transferred from one to another at each frequency. Meanwhile, the estimation of the interaction strength between the selected cryptocurrencies is provided using wavelet cross-spectrum. From the plots represented in Figs. 9, 10 and 11, it can be argued that the cryptocurrency markets were affected by each other during the selected period of the COVID-19 pandemic. The only exception is the market for USDT in which the interaction strengths with BTC, ETH, and LTC were at the lowest level. The heat map from blue to red indicates an increasing strength of correlation between the markets. Blue represents a weak connection, while red denotes a strong relationship among the selected assets. The same pattern applies to the WPS and wavelet coherence plots. One of the most distinguishing features of the evidence from the wavelet cross-spectra is the highest correlation between the selected markets and the others. The results show that the overall relationships of BTC, ETH, and LTC are not limited among themselves but also contain the other markets. However, the scale of that relationship is very decisive among the three even though the cross-correlations from those selected markets are highly significant with the others. From the wavelet cross-spectrum, the spillover between the selected cryptocurrencies was found to be infrequent prior to the second half of October 2020, which was the beginning of the second global lockdown and only exists in the short-run up to 8 days. In the latter period, while the spillovers remain; they become more pronounced along with high frequency. Therefore, from the analyses of conditional volatilities and wavelet cross-spectrum, it can be concluded that the volatility spillover between those markets becomes more significant at the second lockdown compared to the first and it is limited in the short-run.

Results from the three different but integrated models imply that financial investors tended to move to assets where the prices of those assets were biased toward an increase in line with leading a surge in volatility transmission among the cryptocurrencies. Therefore, the economic implication is that the financial investors were substantially exposed to herding behavior during the COVID-19 pandemic. This can be considered as a critical way to understand why and how financial investors follow the same kind of behavior at the time of economic problems that arise from events such as the COVID-19 pandemic or global recessions. As a result of investors’ expectations of potential future earnings during abnormal times, many investors move away from markets where severe financial distress can occur toward different markets such as cryptocurrency markets to compensate for their financial losses. However, the main problem is the potential surge in risky behaviors in these markets, in line with an increase in herding behavior. Since many investors follow the same behavior in these markets, they also initiate an increase in the volatility rate for such assets, in which their prices rose rapidly over a short time.

Given that the volatility spillover was binding for BTC, ETH, and LTC from November 17, 2019, to January 25, 2021, the final step covers the analysis of whether the risky behavior for the crypto assets was valid at the time of the COVID-19 pandemic. Two integrated methods were implemented to identify any possible speculative bubble in selected digital currencies: (1) the *value-at-risk* (VaR) and (2) the *conditional value-at-risk* (CVaR). The technical reason to use those methods is to measure the level of financial risk within cryptocurrencies over the COVID-19 pandemic, and thereby, to formulate an idea about potential explosive behavior. Further, the analysis also integrates Shanghai Composite and S&P 500 indices to compare the weight of different financial instruments in the presence of a speculative bubble. In other words, the potential for loss in different markets is evaluated through the implementation of VaR—which represents a worst-case loss associated with a probability and a time horizon—and CVaR—which is the expected loss if the worst-case threshold is ever crossed—methods, and thus, the possibility of occurrence for defined loss is determined to quantify the level of financial risk. Moreover, these methods control the level of risk exposure in which a surge in risk may presuade some giant investors to invest in that market, which may then result in an increase in the asset price and may tear down the financial markets in what is sometimes referred to as a “crash” or a “bubble burst”.

**Fig. 12:**
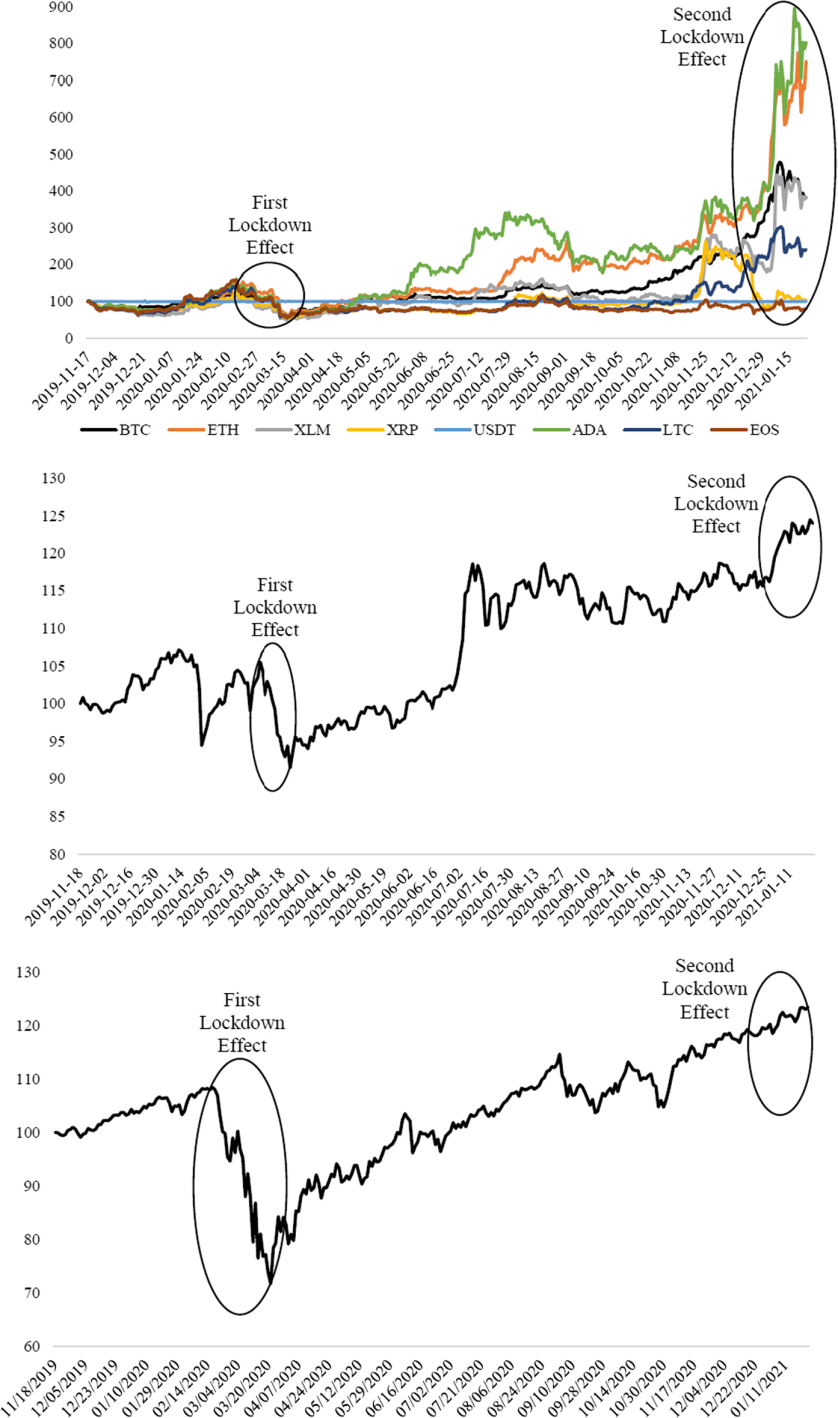
Price changes in Shanghai Stock Exchange, S&P 500, and the Selected Cryptocurrencies. Note: Both the Shanghai Composite Index, S&P 500 Index, and the selected digital currency price series are standardized to a starting price of 100. The Shanghai Composite Index and S&P 500 Index are extracted from Yahoo Finance and Nasdaq, respectively

**Fig. 13:**
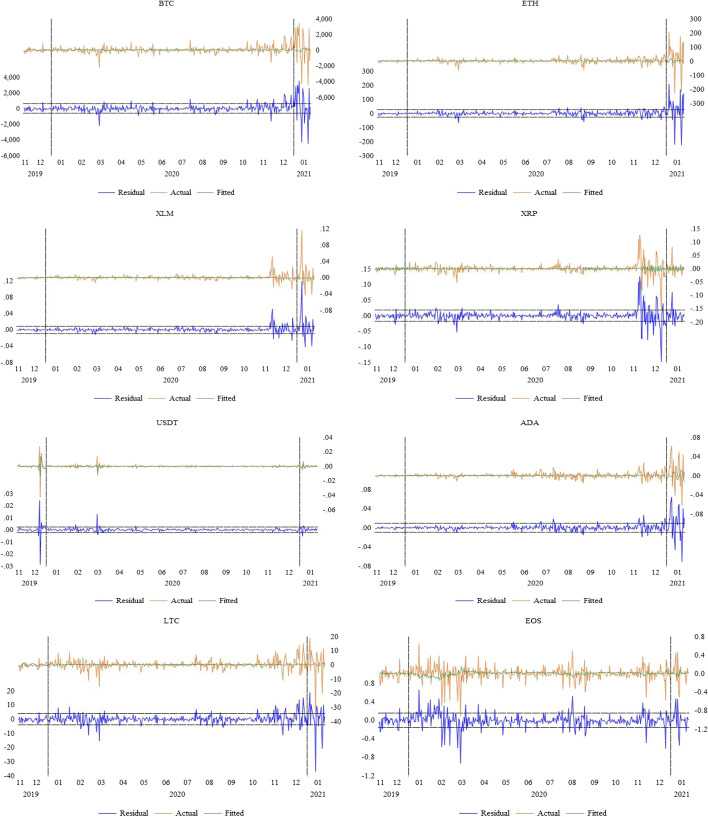
Residual, actual and fitted values. *Source*: CoinDesk, Authors’ calculation

**Fig. 14:**
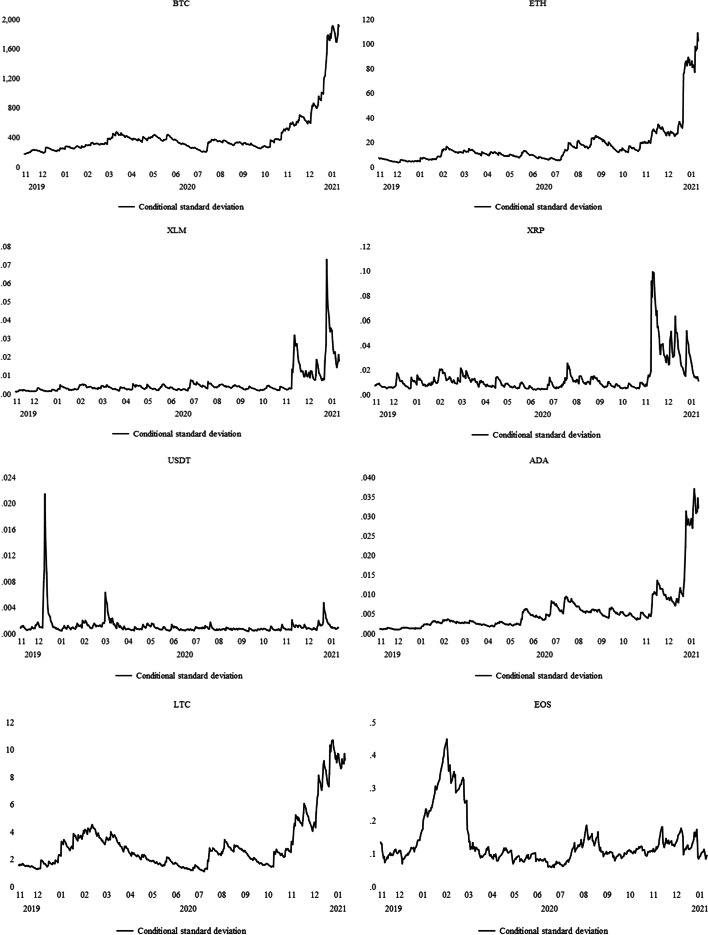
Conditional standard deviations. *Source*: CoinDesk, Authors’ calculation

**Fig. 15:**
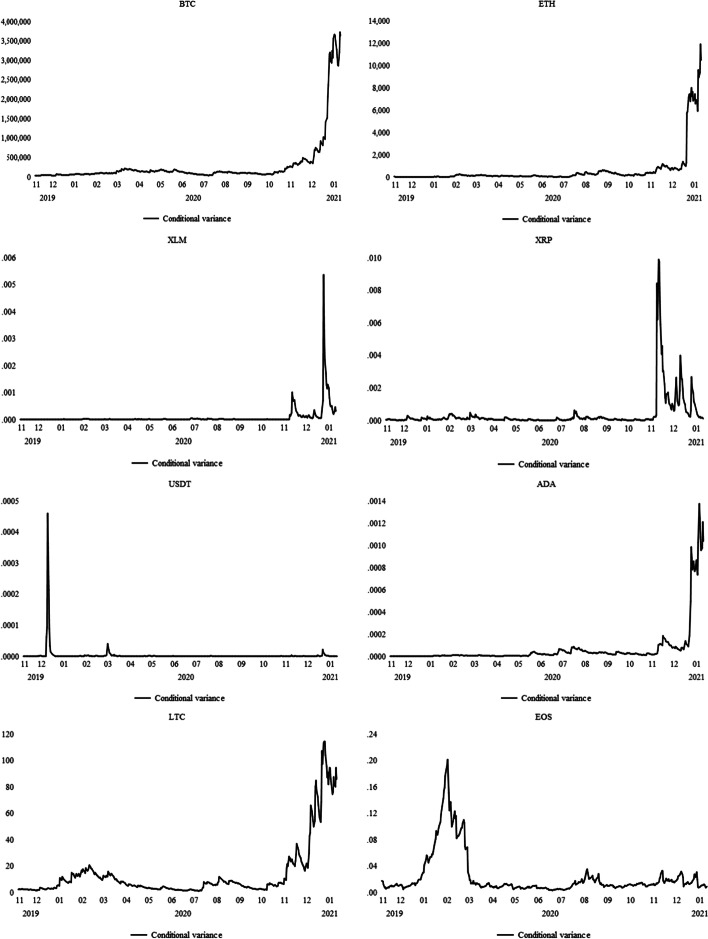
Conditional variances. *Source*: CoinDesk, Authors’ calculation

The first step was to illustrate the safe haven features of eight selected crypto assets in a qualitative manner by analyzing the price dynamics and adding the price changes in the Shanghai Stock Exchange (SSE) and S&P 500 Index. The economic reason for detecting price movement over the selected time horizon was to provide an initial understanding of the safe haven characteristics of these digital currencies. During the decline in the SSE and S&P 500 at the first and second lockdowns, the selected digital assets substantially moved in lockstep with the market, decreasing by more than the SSE and S&P 500 over the two periods as shown in Fig. 12. These facts provide initial evidence that digital currency investments may increase portfolio risk rather than acting as a safe haven resulting in a surge of speculation across the cryptocurrency market.

Table 9 highlights the summary statistics and the VaR and CVaR modeling results for returns corresponding to the SSE, S&P 500, and eight crypto assets. For the selected period of the COVID-19 pandemic, the estimation results show that five digital currencies, BTC, ETH, XLM, ADA, and LTC have higher cumulative returns and larger standard deviations. Over the entire period considered, an investor in these digital assets would have many opportunities to increase their wealth relative to their investments in stock markets. The maximum one-day loss is the highest for the XRP, but the rest of the digital assets, except the USDT, are also relatively high compared with the maximum one-day loss in SSE and S&P 500.

**Table 9:** VaR and CVaR results

	Mean	Standard deviation	Cumulative return	Max One-day loss	VaR (%1)	VaR (%5)	CVaR (%1)	CVaR (%5)
SSE	107.6	8.39	23.9	8.04	− 3.94	− 1.85	− 19.6	− 3.06
S&P 500	103.8	10.3	23.5	12.8	− 7.90	− 3.12	− 10.3	− 5.46
BTC	142.3	82.2	281.2	31.6	− 9.03	− 4.91	− 15.1	− 8.27
ETH	185.3	135.4	651.7	42.3	− 17.5	− 6.65	− 22.3	− 11.9
XLM	125.2	76.9	284.1	27.6	− 15.3	− 7.12	− 17.9	− 11.5
XRP	97.7	37.2	4.68	45.2	− 14.3	− 7.62	− 24.9	− 13.5
USDT	100.1	0.23	0.25	4.2	− 0.46	− 0.17	− 1.49	− 0.50
ADA	212.9	151.6	703.2	34.9	− 14.4	− 8.07	− 19.1	− 12.2
LTC	103.6	47.5	139.9	37.7	− 15.7	− 7.31	− 21.6	− 12.1
EOS	85.2	16.7	19.9	35.2	− 17.9	− 8.14	− 21.6	− 13.3

The VaR and CVaR estimations at the 1% and 5% confidence levels are also shown in Table 9. Regardless of the confidence level investigated, the selected crypto assets, except the USDT, were found to have substantially greater downside risk than SSE and S&P 500. At 5% and 1% confidence levels, the VaR modeling implies that 5% and 1% VaR are the returns for which there are 5% and 1% chances of experiencing a worse return in the sample period, respectively. In this sense, the empirical findings show that both the 5% and 1% VaR are higher for each digital currency, except the USDT, than the SSE and S&P 500, which means that there are 5% and 1% chances of experiencing a return loss than those estimates over the next period in the digital currencies, and that loss in returns would be much higher for digital currencies than the loss in returns for SSE and S&P 500. In addition, the analysis covers the CVaR model to quantify the expected shortfall to measure the likelihood of loss exceeding the value-at-risk. The estimations also indicate the same pattern as that found in VaR modeling, in which financial risk is much higher in digital assets than in stock market indices. Overall, these findings show that investing in selected digital currencies may result in increased financial risk, discrediting the safe haven hypothesis for the cryptocurrency market.

## Concluding remarks

In this study, we examined the return and volatility spillover across eight core cryptocurrencies with the help of the EGARCH model, DCC-GARCH model, and wavelet-based methods. Daily closing prices of those selected cryptocurrencies, namely Bitcoin (BTC), Ethereum (ETH), Stellar (XLM), Ripple (XRP), Tether (USDT), Cardano (ADA), Litecoin (LTC), and Eos (EOS), from November 17, 2019, to January 25, 2021, were used for the analysis. First, we identified Bitcoin as the core market in terms of the demand scale. The EGARCH model was utilized to detect the conditional variance of the closing prices of selected assets and to capture the leverage effects of shocks in terms of the relationship between shocks to variance and shocks to returns. We found that positive shocks had a greater impact on volatility than negative shocks (e.g., the COVID-19 pandemic) of the same magnitude (except for the Tether market,^3^ where the coefficient sign is negative). Therefore, in these markets none of the volatility asymmetries indicates that financial investors are more sensitive to positive news (i.e., the non-persistence of the COVID-19 pandemic in the near future or no lockdown process over the globe) than they are to negative ones.

This study used the DCC-GARCH model to identify the effects of the presence of relevance for capturing the volatility spillovers in the cryptocurrency markets during the COVID-19 outbreak. The DCC-GARCH model results show high volatility spillover across three return pairs (i.e., Bitcoin, Ethereum, and Litecoin), while it indicates the possibility of moderate and close to low volatility spillover for the rest of the return pairs. The maximum likelihood estimates of the Gaussian DCC model of cryptocurrencies show that volatilities could be mainly explained by their fluctuations. Further, the correlation structure between the selected asset pairs strengthened during the moment of shocks, especially for Bitcoin, Ethereum, and Litecoin prices, implying investor panic. This means that the COVID-19 pandemic has led to more integrated cryptocurrency markets, and thus has also stimulated herding behavior among financial investors.

The final step was to apply the multiscale correlation technique using wavelet methods, which capture information across different frequencies without losing information from the given time horizon. Using these methods provided a way to investigate the relationship between various assets at different time scales and frequency bands by capturing the low- and high-scale effects of any shock that occurred within and across financial markets. We examined three types of wavelet methods, namely wavelet power spectrum, wavelet coherence, and wavelet cross-spectrum. The common point of these measures is that the possibility of the selected cryptocurrency markets to a large extent is significant in the short run, especially as depicted in the wavelet coherence analysis. In addition, from the wavelet cross-spectrum, it is found that volatility spillover is relatively high for three major cryptocurrency markets, namely Bitcoin, Ethereum, and Litecoin, which was exacerbated after the second lockdown effect in November 2020, but persisted up to 128 days. However, the others reflected a moderate spillover in terms of their volatility and existed only in the short run, up to 8–16 days.

The empirical findings imply that cryptocurrency markets are largely assumed to be one of the core financial platforms where financial investors increasingly participated for higher returns during the COVID-19 outbreak. However, it led to three major outcomes: (1) increased levels of risky investments, (2) a greater level of herding in financial markets, and (3) exacerbating the nature of volatility spillover. While a moderate level of volatility offers a certain number of advantages to financial investors to diversify their assets, it poses some problems because the cryptocurrencies have no intrinsic value, and they do not offer any dividends or a specific returns. Therefore, these markets face notable concerns over circumstances such as legal position, safety, and transparency. Considering the apprehension about the ongoing increase in the prices of such cryptocurrencies together with unfettered demand, the increasing uncertainty and the level of risk may cause the dynamics of volatility spillover to change over time. In conclusion, increased unfettered demand for some major cryptocurrencies may lead to a serious loss of returns for many financial investors, resulting in a sudden unexpected decrease in prices.

Although the empirical findings confirm that the volatility spillover in cryptocurrency markets is a widespread issue, there are some limitations to this study. Primarily, the methodological framework restricts the implementation of additional factors in empirical analysis. Therefore, following the general trend in the existing literature, empirical estimations are limited to some degree. In addition, although the presence of volatility spillover for the three major cryptocurrencies Bitcoin, Ethereum, and Litecoin are validated under the implementation of current methods, the reasons behind the volatility spillover can be estimated based on observations. This fact can also be generalized for other cryptocurrency markets used in the empirical analysis. Another critical limitation concerns the existing literature in which the studies are not extensive in terms of their role of exploring explosive behaviors on the volatility of digital asset markets during the COVID-19 pandemic. This restricts us from comparing the empirical results found in this study with those of others. Finally, the data selection process did not proceed with rule-bound data because the series were selected based on a comparison technique by looking at alternative data. However, even though the data selection process did not adhere to the rules, the empirical findings pursuant to the use of prices of selected cryptocurrencies that are derived from the alternative datasets validate the current results in the context of robustness checks. In light of these limitations and drawbacks, potential future directions for research are based on two things: first, future studies will be expanded using alternative methodologies, considering the presence of speculative-led behaviors and volatility spillovers in cryptocurrency markets during the COVID-19 pandemic. Second, future studies will use alternative models in which other volatility-based potential factors can be integrated into the analysis to detect reasons for a surge in speculative motives among investors and thereby volatility spillover.

## Data Availability

The datasets used and/or analyzed during the current study are available from the corresponding author on reasonable request.

## Appendix 1: Residual, actual, and fitted values

See Fig. 13.

## Appendix 2: Conditional standard deviations

See Fig. 14.

## Appendix 3: Conditional variances

See Fig. 15.

## Abbreviations

ADA: Cardano
ADF: Augmented Dickey–Fuller
AIC: Akaike information criterion
AR: Autoregressive
ARCH: Autoregressive conditional heteroskedasticity
ARCH-LM: Autoregressive conditional heteroskedasticity-Lagrange multiplier
BEKK: Baba–Engle–Kraft–Kroner
BHHH: Berndt–Hall–Hall–Hausman
BTC: Bitcoin
CVaR: Conditional value-at-risk
DCC-GARCH: Dynamic conditional correlation-generalized autoregressive conditional heteroskedasticity
DW: Durbin–Watson
EGARCH: Exponential generalized autoregressive conditional heteroskedasticity
EOS: Eos
ETH: Ethereum
GARCH: Generalized autoregressive conditional heteroskedasticity
HAR: Heterogeneous autoregressive
IGARCH: Integrated generalized autoregressive conditional heteroskedasticity
LM: Lagrange multiplier
LTC: Litecoin
MGARCH: Multivariate generalized autoregressive conditional heteroskedasticity
MIDAS: Mixed-data sampling
ML-ARCH: Maximum likelihood-autoregressive conditional heteroskedasticity
MS-VARX: Markov regime-switching vector autoregressive with exogenous variables
MSV: Multivariate stochastic volatility
OECD: Organisation for economic cooperation and development
OPG: Outer product of gradients
SIC: Schwarz information criterion
SSE: Shanghai stock exchange
USDT: Tether
XLM: Stellar
XRP: Ripple
WPS: Wavelet power spectrum
VaR: Value-at-risk

## Footnotes

1 For more information on opinion dynamics in finance and business, please see Zha et al. (2021).

2 Following the arguments proposed by Alexander and Dakos (2020), the robustness check for the analysis using alternative data derived from different data sources such as Goldprice, CoinMarketCap, Coinbase, and Investing was implemented towards the use of same models. However, the results were not changed to a large extent. Three cyrptocurrencies (i.e., Bitcoin, Ethereum, and Litecoin) were the major ones that they were exposed to explosive behavior by the financial investors at the COVID-19 pandemic. In that vein, the study is based on Coindesk data since the results were not substantially changed. The second reason to select Coindesk data is based on the fact that this data source instantly reveals the change in prices of cryptocurrencies and thus it transmits a huge amount of information for the researchers.

3 This study considers one of the effective stablecoins, namely the Tether, to show that the valuations of stablecoins are supposed to be less volatile than other digital assets, since they are pegged directly to a fixed, non-virtual currency. Therefore, in consideration of the empirical findings, it can be argued that if the most volatile cryptocurrencies are pegged to an economically and financially strong economic units, some major problems that *may* emerge in those markets can be slightly decreased in the long-run.
